# Identifying subjects at risk of liver cirrhosis via a range of thresholds for common fibrosis markers: A Welsh general population‐based cohort study

**DOI:** 10.1111/joim.70064

**Published:** 2026-01-07

**Authors:** Trevor A. Hill, Joe West, Joanne R. Morling, Colin J. Crooks

**Affiliations:** ^1^ Translational Medical Sciences, School of Medicine, University of Nottingham Nottingham UK; ^2^ Gastrointestinal and Liver Theme National Institute for Health Research (NIHR) Nottingham Biomedical Research Centre (BRC) Nottingham University Hospitals NHS Trust and the University of Nottingham, School of Medicine, Queen's Medical Centre Nottingham UK; ^3^ Lifespan and Population Health, School of Medicine, University of Nottingham Nottingham UK; ^4^ Department of Clinical Medicine Aarhus University Aarhus Denmark

**Keywords:** decision support techniques, diagnosis, epidemiology, fibrosis, hepatocellular carcinoma, liver diseases

## Abstract

**Background:**

Liver disease is on the increase worldwide, with cirrhosis and liver cancer accounting for around 3.5% of all deaths.

**Objectives:**

Investigate the prognostic utility of three non‐invasive liver fibrosis markers in the Welsh primary care population for identification of those at risk of cirrhosis or hepatocellular carcinoma (HCC).

**Methods:**

Using the Secure Anonymised Information Linkage (SAIL) Databank at Swansea University (2000–2017), we identified people with liver blood tests allowing calculation of three commonly used liver fibrosis markers: aspartate transaminase to alanine transaminase (AST/ALT) ratio, AST to platelet ratio index (APRI) and fibrosis‐4 index (FIB‐4). We modelled 10‐year risk of cirrhosis/HCC across a range of thresholds using competing risk survival analysis and compared their prognostic value using decision curve analysis (DCA).

**Results:**

Blood tests enabling calculation of FIB‐4, APRI and AST/ALT were available for 203,005 people. At commonly utilized cut‐points to detect advanced fibrosis/cirrhosis of 3.25, 1.5 and 1.0 for FIB‐4, APRI and AST/ALT, respectively, the 10‐year risks of cirrhosis/HCC were 4.7%, 16% and <1%. DCA demonstrated the APRI has the greatest net benefit for estimating cirrhosis/HCC risk over 10 years, in a general population compared to AST/ALT or FIB‐4. In higher risk subgroups, a greater proportion of at‐risk patients were captured for fewer referrals. This was also observed in groups with combinations of risk factors.

**Conclusion:**

At risk thresholds often used for referral, liver fibrosis markers had prohibitively high false positive rates unless restricted to subgroups at increased risk of developing severe liver disease.

## Graphical abstract

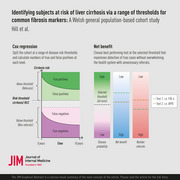

**Abbreviations**
ALT: alanine transaminase
APRI: aspartate to platelet ratio index
AST: aspartate transaminase
AST/ALT: aspartate transaminase to alanine transaminase ratio
DCA: decision curve analysis
FIB‐4: fibrosis‐4 index
GGT: gamma‐glutamyl transferase
HCC: hepatocellular carcinoma
MASLD: metabolic dysfunction‐associated steatotic liver disease
SAIL: Secure Anonymised Information Linkage Databank

## Introduction

Liver disease is on the increase worldwide, with cirrhosis and liver cancer accounting for around 3.5% of all deaths. Yet identifying people at risk of severe liver disease early enough in their disease trajectory to enable intervention has proved difficult [1, 2, 3]. Population screening or targeted case‐finding in at‐risk groups, via the use of non‐invasive liver fibrosis markers (e.g., blood markers), has been proposed to address this problem [4, 5] as earlier detection has the potential to save lives and could be implemented in many health care systems [6].

Over the years, routinely collected indirect markers such as the enzymes alanine transaminase (ALT) and aspartate transaminase (AST) have been combined with other factors to improve their ability to predict the occurrence of future liver disease [7, 8]. However, there has never been an assessment in a large, representative, general population of the prognostic utility of these markers at their reported cut‐points for identifying severe liver disease. Specifically, they have not been compared to one another in terms of their ability to accurately predict the onset of cirrhosis or hepatocellular carcinoma (HCC) nor has the variation in risk of cirrhosis/HCC at different cut‐points or performance within specific at‐risk subgroups been quantified.

To fill this gap in knowledge, we used data from almost the entire Welsh population and employed decision curve analysis (DCA) [9, 10] to directly compare the performance of the AST to platelet ratio index (APRI) [11], AST/ALT ratio [12] and fibrosis‐4 index (FIB‐4) [13]. Using DCA allows the presentation and direct comparison of the prognostic performance of the markers at a range of clinically reasonable risk thresholds not previously reported in a general population sample. This information is needed to aid a decision regarding the best marker for case‐finding purposes, at whichever risk threshold, or range of thresholds, might be used as the optimal intervention strategy.

## Methods

### Population

We identified all adults (≥18 years) for the period January 2000 to December 2017 from the Welsh Longitudinal General Practice (WLGP) dataset stored on the Secure Anonymised Information Linkage (SAIL) Databank at Swansea University [14, 15]. Patients were selected with one or more liver‐related blood serum tests via a list of Read codes (Table S1), where the blood tests were recorded in follow‐up time defined by periods of GP registration [16]. SAIL data include over three quarters of Welsh general practices and have been shown to be representative of the Welsh population in terms of sex, age and deprivation [17]. Our previous article [18] describes in more detail how we obtained the original cohort for analysis.

Individual patient data are linked to the Annual District Death Extract (ADDE), the Patient Episode Database for Wales (PEDW), the Welsh Cancer Intelligence and Surveillance Unit (WCISU) and the Welsh Demographic Service Dataset (WDSD). This enabled us to identify subjects with the outcome of interest (cirrhosis/HCC), and periods of the study in which subjects were registered with a Welsh GP.

### Cohort selection

**Fig. 1:**
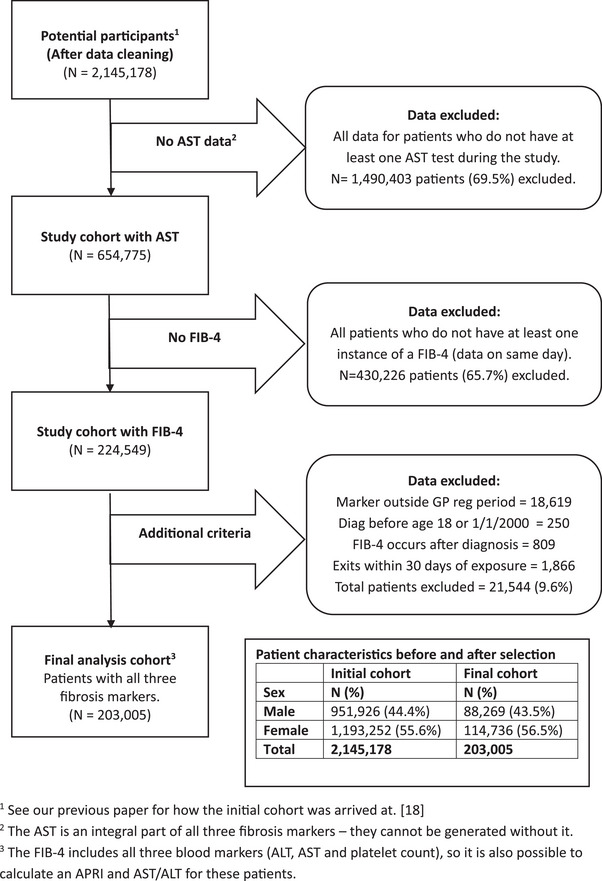
Flowchart showing how the analysis file for patients with all three markers, was generated. ^1^See our previous paper for how the initial cohort was arrived at ref. [18]. ^2^The aspartate transaminase (AST) is an integral part of all three fibrosis markers—they cannot be generated without it. ^3^The fibrosis‐4 index (FIB‐4) includes all three blood markers (alanine transaminase [ALT], AST and platelet count), so it is also possible to calculate an AST to platelet ratio index (APRI) and AST/ALT for these patients.

We restricted the cohort to patients for whom we could calculate a FIB‐4 (and hence an APRI and AST/ALT) to ensure the time at risk was the same for the three markers, for each subject in the analysis. See Fig. 1 for a summary of the analysis cohort derivation and Fig. S1 for the study design. Where patients had multiple exposures (i.e., more than one FIB‐4 or APRI), the first instance of the marker, regardless of whether it was abnormal or normal, was used in the analysis. We omitted all patients with an exit date due to a diagnosis of cirrhosis, HCC, or loss to follow up within 30 days of the blood test marker date. This was to avoid modelling blood tests taken as part of the end point diagnosis. Follow up began 30 days after the blood test marker date.

### Identification of cirrhosis and hepatocellular carcinoma outcomes

Severe liver disease was defined as cirrhosis and/or HCC. These were identified using the recently published cirrhosis consensus code list [19] plus codes for HCC. As the consensus list contained only ICD10 codes, we mapped Read v2 and OPCS4 codes to allow use in GP and hospital procedure data. Our final list contained additional Read codes for cirrhosis, alcoholic cirrhosis, oesophageal varices and portal hypertension, from the GP record, together with OPCS4 codes for operations on oesophageal varices from secondary care. See Table S2 for the full list of codes used.

### Identification of risk factors

We obtained information on risk factors from the Welsh GP event file on SAIL for diabetes, obesity and hazardous alcohol use [20]. Diabetic cases, both Types I and II, were defined using combinations of Read v2 code lists utilized by a recent study [21] and supplemented by searching the Welsh GP event file for similar codes (Table S3).

Diagnoses of hazardous alcohol use were defined using Read v2 codes from recent studies [22, 23], omitting codes indicating acute or accidental poisoning with alcohol. We supplemented this with codes for dependent and non‐dependent alcoholism in remission, alcohol support services and the number of units consumed per week ≥14 U, as per recent UK guidelines [24] (Table S4).

BMI and obesity were defined using Read v2 codes from the HDRUK phenotype library [25, 26], and through the calculation of BMI from height and weight data with a BMI ≥ 30 indicating obesity (Table S5). The height reading was allowed to precede the weight reading. Where a patient had more than one BMI value present on the same date the average was calculated.

Patients were identified as having one or more of these risk factors if the factor was recorded before and up to 30 days after the entry date to the study (date of fibrosis marker exposure).

### Cox model setup

Subjects were censored at the earliest of their time of death, at the end of the study analysis period (31 December 2017) or at the point their GP registration period ended. If data suggested they died or experienced the outcome after the GP registration period, the subject was censored at the date their GP registration ended. We took this approach as we could not be confident time and events outside GP registration periods were recorded accurately and to avoid assigning an incorrect status to a subject. If the subject died on the same date as a diagnosis of cirrhosis/HCC the outcome took priority over death in the analysis; that is, they were included as having an outcome, not as being censored.

### Statistical analysis

We explored the relationship between exposure and outcome by Cox survival models utilizing fractional polynomials, with the transformation selected using the default closed‐test algorithm which employs a backwards elimination method [27, 28]. We preserved the continuous nature of the predictor variables (the marker scores) rather than stratifying the risks via a binary exposure and calculated results at several different risk thresholds as per recent recommendations [29].

We calculated 10‐year risk of cirrhosis in the presence of the competing risk of death by running separate cause‐specific models for cirrhosis and death and combining the hazard contributions to calculate competing risk‐adjusted cumulative incidences from these via a widely utilized method [30].

The predicted cirrhosis and HCC free survival from these Cox models was used to derive 10‐year cirrhosis risk across the range of potential liver marker cut offs. The observed cirrhosis and HCC free survival were calculated using Kaplan–Meier estimates above and below these risk‐estimate thresholds and used to derive the corresponding true and false, positive and negative predictive values. The net benefit was also calculated, which is a weighted difference between true and false positives, and was plotted as a decision curve to visually compare individual liver markers via the method introduced by Vickers [9, 10].

The decision curve shows net benefit on the *y*‐axis, with the risk threshold (or probability of disease) shown on the *x*‐axis. The probability of disease, cirrhosis/HCC in this case, is obtained at each risk threshold from the Cox regression model. Implementing a threshold for referral at a cirrhosis risk of 10% implies accepting 9 unnecessary referrals to find 1 cirrhosis/HCC case. Net benefit is a summary measure which shows the value of applying the threshold for referral at a range of disease risk levels, taking the true positive proportion and a weighted false positive proportion into account. The higher the net benefit value at any specific risk level, the better that approach. The DCA curve allows direct comparison of different diagnostic methods, or fibrosis markers in our example, at a range of disease risk levels that would reasonably be considered for clinical intervention. The plot also includes two default approaches, for referring all patients and referring none.

We also compared standard diagnostic measures (sensitivity/specificity) for each marker, and for a representative population of 100,000 people, the number of referrals that would be made, the number of referrals that could potentially be avoided, the number of cirrhosis cases correctly predicted, and the number of cirrhosis cases not predicted at each threshold compared to an investigate‐all strategy.

Diagnostic measures were calculated at 10 years from the survival probabilities obtained from the Cox model. Subjects with less than 10 years of follow up were included in the calculation and counted as cases if they experienced the outcome in this time. Subjects who experienced the outcome after 10 years were censored at 10 years. Patients were assigned to the category (either true or false positive or true or false negative) depending on whether they experienced the outcome within 10 years and whether their marker score was above or below the current threshold being considered.

Diagnostic quantities such as sensitivity and specificity were generated by first calculating the true and false, positive and negative proportions at each of the biomarker thresholds being considered, from the values for cirrhosis and HCC free survival, taken from the survival model.

Finally, we repeated the above process for each risk factor subgroup and their combinations, updating the risk probabilities for each threshold by re‐running the Cox models in each subgroup. We calculated the numbers of referrals, cases detected and missed and unnecessary referrals per the number of subjects who would be expected to have each risk factor in each 100,000 of the population. Additionally, we carried out an age‐stratified analysis, repeating the main analysis in subjects aged <65 years and those ≥65 years of age, categories that have been used previously to investigate prediction of fibrosis via FIB‐4 score in patients with MASLD [31].

We also carried out several cause‐specific sensitivity analyses, examining the relationship between the markers and the risk of cirrhosis/HCC only, without the competing risk of death. These included re‐running the main analysis, an age‐stratified analysis, evaluating the discriminatory power of the models over time, examining the effect of restricting the outcome to symptomatic outcome codes and varying the length of the exclusion period. In addition, we calculated 10‐year incidence rates and cumulative incidence of cirrhosis/HCC for both the analysis cohort and the patients without a FIB‐4 who were excluded from the main analysis for comparison.

Model checking included global proportional hazards (PH) tests, plots of Schoenfeld residuals, counts of critical, scaled DFBETA values and component‐plus‐residual plots to view the final model fit. Table S6 in the supplement summarizes the results of the PH tests and counts of scaled DFBETAS for the cirrhosis models. The results for the death outcome are not shown; however, the pattern of results is very similar. Analyses were carried out in Stata SE v18.0 (StataCorp, TX, USA).

### Patient and public involvement

Patients were not involved in this study.

## Results

### Patient characteristics

After restricting the cohort to patients with all three markers (Fig. 1), there were 203,005 patients remaining for analysis (Table 1). Median (IQR) age when tested was 56 (41–70) years. Overall, 43.5% of patients (*n* = 88,269) were male. Males had higher FIB‐4 (1.03 vs. 0.90) and APRI scores (0.25 vs. 0.19) than females. There was a greater proportion of high APRI and FIB‐4 scores using routine thresholds in males (1.7% vs. 1.0% and 3.0% vs. 1.9%, respectively); however, there were more females with high AST/ALT ratios (2.7% vs. 4.6% at the ≥2.0 cut‐point). The median (IQR) time from initial exposure to diagnosis was 4 (1–8) years. For those not diagnosed with cirrhosis, median time from initial exposure to censoring was 9 (4–12) years. There were 2192 cirrhosis/HCC cases identified in the 18‐year study period of which 1854 (85%) occurred within GP registration periods and were available to the analysis.

**Table 1:** Demographic characteristics for patients with all three markers (N = 203,005).

	All	Male	Female
*N* patients	203,005 (100.0%)^a^	88,269 (43.5%)	114,736 (56.5%)
Age when tested			
18–39	45,279 (22.3%)	17,292 (19.6%)	27,987 (24.4%)
40–59	69,095 (34.0%)	32,060 (36.3%)	37,035 (32.3%)
60–79	67,838 (33.4%)	31,655 (35.9%)	36,183 (31.5%)
80+	20,793 (10.2%)	7262 (8.2%)	13,531 (11.8%)
Deprivation quintile			
Most deprived	33,722 (16.6%)	14,830 (16.8%)	18,892 (16.5%)
Next most deprived	45,677 (22.5%)	19,896 (22.5%)	25,781 (22.5%)
Median deprivation	47,273 (23.3%)	20,398 (23.1%)	26,875 (23.4%)
Next least deprived	48,535 (23.9%)	21,276 (24.1%)	27,259 (23.8%)
Least deprived	24,848 (12.2%)	10,508 (11.9%)	14,340 (12.5%)
Missing	2950 (1.5%)	1361 (1.5%)	1589 (1.4%)
Diagnosed with cirrhosis^b^			
Yes, within GP reg period	1854 (0.9%)	1092 (1.2%)	762 (0.7%)
Yes, outside GP reg period	338 (0.2%)	214 (0.2%)	124 (0.1%)
No	200,813 (98.9%)	86,963 (98.5%)	113,850 (99.2%)
Died in study^b^			
Yes, within GP reg period	42,517 (20.9%)	19,776 (22.4%)	22,741 (19.8%)
Yes, outside GP reg period	7694 (3.8%)	3438 (3.9%)	4256 (3.7%)
No	152,794 (75.3%)	65,055 (73.7%)	87,739 (76.5%)
Median (IQR) marker values			
ALT (IU/L)	23 (16–35)	28 (19–42)	20 (14–29)
AST (IU/L)	22 (18–28)	24 (20–31)	21 (17–26)
Platelet count (×10^9^/L)	262 (221–311)	245 (208–290)	275 (234–324)
APRI^c^	0.21 (0.16–0.30)	0.25 (0.19–0.34)	0.19 (0.14–0.25)
FIB‐4	0.95 (0.62–1.42)	1.03 (0.69–1.51)	0.90 (0.58–1.34)
AST/ALT	1.00 (0.72–1.29)	0.89 (0.67–1.17)	1.06 (0.78–1.36)
High marker values			
APRI ≥ 1.5	2696 (1.3%)	1554 (1.7%)	1152 (1.0%)
FIB‐4 ≥ 3.25	4823 (2.4%)	2668 (3.0%)	2155 (1.9%)
AST/ALT ≥ 1.0	102,575 (50.5%)	35,951 (40.7%)	66,624 (58.1%)
AST/ALT ≥ 2.0	7668 (3.8%)	2386 (2.7%)	5282 (4.6%)
Risk factors^d^			
Diabetes	17,784 (8.8%)	9745 (11.0%)	8039 (7.0%)
Obesity	47,818 (23.6%)	20,296 (23.0%)	27,522 (24.0%)
Hazardous alcohol use	29,139 (14.4%)	21,468 (24.3%)	7671 (6.7%)

Abbreviations: ALT, alanine transaminase; APRI, AST to platelet ratio index; AST, aspartate transaminase; FIB‐4, fibrosis‐4 index.

^a^ A total of 21,544 patients who have a FIB‐4 have been omitted due to either being diagnosed with cirrhosis before age 18 or before 1 January 2000, the earliest available marker falling outside a GP registration period, the earliest available marker occurring after the diagnosis of cirrhosis, or they exit the study within 30 days of their exposure date.

^b^ Only outcomes diagnosed within a GP period are included in the analysis.

^c^ The APRI has been calculated using an ULN of 40 for all patients.

^d^ Only risk factors occurring before the exposure date or within 30 days after exposure are included in the analysis.

### Absolute rates and cumulative incidence of cirrhosis/HCC

Table S7 shows the observed cumulative incidence of cirrhosis/HCC, taking death as a competing risk into account, above each standard cut‐point of the fibrosis markers. However, the risk over the range of thresholds was not linear (Fig. S2). Therefore, fractional polynomial transformations were fitted as described in the methods. To allow other groups to reproduce our work, the transformations selected are shown in Table S8.

### Decision curve analysis

**Fig. 2:**
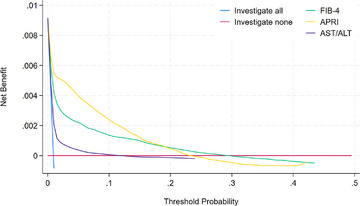
Decision curve for the main cohort (N = 203,005 patients).

Figure 2 presents the decision curves for the three markers. These were calculated in the main cohort from the predicted survival estimates from the Cox models fitted with fractional polynomial transformations and take account of the competing risk of death.

Table S9 shows net benefit and diagnostic test accuracy measures for risk of 10‐year cirrhosis/HCC for each marker at risk threshold probabilities of 1%, 2%, 3%, 10% and 20%.

In the general population, the APRI and FIB‐4 performed better than the AST/ALT (have higher net benefits) across all threshold probabilities for cirrhosis. Table S9 shows the common cut‐point value for the FIB‐4 (3.25) corresponds to a much lower cirrhosis/HCC threshold (4.7% vs. 16%) than employed by the APRI (1.50). Figure 2 shows that below a risk of 18% then APRI is the preferred strategy, when more than 5–6 unnecessary referrals are made for each cirrhosis case diagnosed. If less unnecessary referrals are preferred, that is, a risk threshold over 18%, then FIB‐4 appears to be the better strategy.

### Application of markers per 100,000 population screened

The performance of the markers, including numbers of referrals, at selected thresholds extrapolated to 100,000 people is shown in Table 2. Higher risk thresholds for referral invariably mean less false positive patients referred but a corresponding reduction in sensitivity of predicting cirrhosis cases. However, at each risk threshold for referral there were differences in performance between the liver markers; at risks of 1% the APRI results in fewer overall referrals, substantially more cases identified and fewer unnecessary referrals. Although the APRI detects a higher proportion of cases at all risk levels compared to the FIB‐4, at higher risks of cirrhosis/HCC of 2%–20% the FIB‐4 results in fewer overall referrals, fewer unnecessary referrals and at risks of 10%–20% a higher proportion of true cases with respect to overall number of referrals. The AST/ALT is outperformed by the other two tests.

**Table 2:** Extrapolating^a^ true and false positive and negative rates from the cox model to 100,000 liver blood tests.

	Liver marker models				
Cirrhosis risk threshold^b^	No. referrals made for patients above predicted threshold	No. detected cirrhosis cases (true positives)	No. missed cirrhosis cases (false negatives)	True negatives (non‐cases)	No. unnecessary referrals (false positives)
FIB‐4					
0% (refer everyone)	100,000	950 (100%)	0	0	99,050
1%	22,260	640 (67.4%)	310	77,440	21,620
2%	8490	500 (52.6%)	450	91,070	7990
3%	4620	420 (44.2%)	530	94,860	4190
10%	910	220 (23.2%)	730	98,360	690
20%	410	130 (13.7%)	820	98,770	280
APRI					
0% (refer everyone)	100,000	950 (100%)	0	0	99,050
1%	19,020	760 (80.0%)	190	80,790	18,270
2%	9110	720 (75.8%)	230	90,660	8400
3%	6310	670 (70.5%)	280	93,420	5640
10%	2110	420 (44.2%)	530	97,370	1680
20%	1030	230 (24.6%)	720	98,260	800
AST/ALT					
0% (refer everyone)	100,000	950 (100%)	0	0	99,050
1%	25,380	420 (44.2%)	530	74,090	24,960
2%	4820	190 (20.0%)	760	94,420	4630
3%	2030	130 (13.7%)	820	97,160	1900
10%	290	40 (4.2%)	910	98,800	250
20%^c^	80	10 (1.1%)	940	98,990	70

Abbreviations: ALT, alanine transaminase; APRI, AST to platelet ratio index; AST, aspartate transaminase; FIB‐4, fibrosis‐4 index.

^a^ The Cox model takes account of censoring and calculates more cases than are diagnosed in the cohort. Numbers are estimates and have been rounded to the nearest 10. As a consequence, the total number of subjects may not be exactly 100,000 on some rows.

^b^ The cirrhosis/HCC risk takes account of competing risk of death.

^c^ At high risk levels the model for AST/ALT not calculable as there are very few cases. The actual risk level utilized here (and the highest risk according to the marker) was ∼19%.

### Risk factor subgroups

**Fig. 3:**
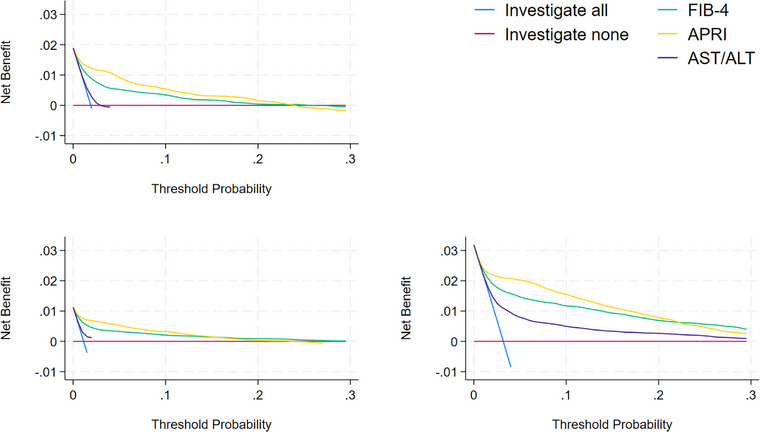
Decision curve analysis (DCA) plots for the diabetic patients (top left), the obese patients (bottom left) and those with hazardous alcohol use (bottom right) compared.

There were 47,818 (23.6%) patients identified as obese/BMI > 30, 17,784 (8.8%) patients identified as diabetic and 29,139 (14.4%) identified with a history of hazardous alcohol use. Figure 3 shows the increased net benefit for the liver markers in all three subgroups, with the highest net benefit across all threshold probabilities in the subgroup with a history of hazardous alcohol use.

Table 3 shows a comparison of number of referrals and cases detected and missed that would be expected in every 100,000 subjects, using a risk threshold of 3% in the main cohort, compared with different combinations of risk group. As the underlying risk of cirrhosis increased in the subgroup, at the same risk threshold for referral and corresponding false positive rate there was an increase in the sensitivity of correctly predicting cirrhosis. This increase in sensitivity was greatest for APRI in every subgroup. There was a similar pattern in performance for each liver marker at the 1% and 10% thresholds for referral (Tables S10 and S11).

**Table 3:** Possible case‐finding strategies for a 3% risk of cirrhosis/hepatocellular carcinoma (HCC).

Population (*N* screened)	Marker	Marker score	No. referrals	Cases detected (%)^a^	Cases missed	Total cases	Unnecessary referrals
Main cohort (*N* = 100,000)							
	APRI	0.54	6310	670 (70.5)	280	950	5640
	FIB‐4	2.60	4620	420 (44.2)	530	950	4190
	AST/ALT	2.21	2030	130 (13.7)	820	950	1900
Obese patients (*N* = 24,000)							
	APRI	0.50	2380	230 (82.1)	50	280	2160
	FIB‐4	2.06	1910	160 (57.1)	120	280	1750
	AST/ALT	1.78	650	40 (14.3)	240	280	610
Hazardous alcohol use (*N* = 14,000)							
	APRI	0.39	3370	390 (86.7)	60	450	2980
	FIB‐4	1.51	3650	340 (75.6)	110	450	3310
	AST/ALT	1.18	4040	280 (62.2)	170	450	3760
Diabetic patients (*N* = 8800)							
	APRI	0.41	1641	170 (94.4)	10	180	1475
	FIB‐4	1.95	1455	110 (61.1)	70	180	1342
	AST/ALT	1.37	1080	40 (22.2)	140	180	1040
Obese with diabetes (*N* = 4200)							
	APRI	0.39	1070	110 (100)	0^b^	110	960
	FIB‐4	1.67	930	80 (72.7)	30	110	840
	AST/ALT	1.03	1120	50 (45.5)	60	110	1070
Obese and hazardous alcohol use (*N* = 3900)							
	APRI	0.43	970	100 (90.9)	10	110	860
	FIB‐4	1.52	960	90 (81.8)	20	110	870
	AST/ALT	1.04	930	60 (54.5)	50	110	870
Diab. and hazardous alcohol use (*N* = 1400)							
	APRI	0.34	560	60 (100)	0^b^	60	500
	FIB‐4	1.47	540	60 (100)	0^b^	60	480
	AST/ALT	0.85	690	50 (83.3)	10	60	640
All three risk factors (*N* = 700)							
	APRI	0.31	370	40 (100)	0^b^	40	330
	FIB‐4	1.31	330	40 (100)	0^b^	40	290
	AST/ALT	0.76	380	40 (100)	0^b^	40	350

Abbreviations: ALT, alanine transaminase; APRI, AST to platelet ratio index; AST, aspartate transaminase; FIB‐4, fibrosis‐4 index; NB, numbers take account of competing risk of death.

^a^ The percentage is out of the total number of cases.

^b^ Due to small numbers these cells have been set to zero in the above table. Note also that we cannot claim our model counts to be accurate down to the single subject, so all counts have been rounded to the nearest 10. As a result, some row totals may be slightly out by 10. This has not affected comparisons between the markers in each risk group, however.

Table S12 shows how the pseudo‐populations were calculated for Table 3, Tables S10 and S11. Note that as our method relies on comparing survival estimates, and the models are not perfect, small counts <5 should be treated as equal as we cannot claim our method is accurate down to the single subject. As a result, we have rounded all counts to the nearest 10 in these three tables.

### Sensitivity analyses

Compared to a cause‐specific analysis, taking account of the competing risk of death reduced the performance of all three markers. Table S13 presents the cause‐specific analysis utilizing the cirrhosis/HCC outcome only at the 3% risk level for comparison with Table 3 and suggests the APRI is affected least, with a smaller reduction in the proportion of cases detected compared to the other markers. DCA plots confirmed the net benefit of all three markers is also reduced.

For the age‐stratified analysis, we found the net benefit of all three markers was slightly inferior in patients aged ≥65 years compared to those aged <65 years. Figure S3 presents DCA plots comparing the two groups. Table S14 shows the risk thresholds associated with commonly applied marker scores, in the two age groups. In general, the risk threshold for cirrhosis/HCC is lower in the older age group.

Table S15 shows Harrell's C‐statistic [32] for different lengths of follow‐up time. The results show that the APRI is least affected, and although there is a slight drop in performance, has a consistent discriminatory power across the whole period of follow‐up time. The FIB‐4 also performs well, but the AST/ALT has relatively poor performance.

Table S16 and Fig. S4 present the results of the analysis restricted to symptomatic cirrhosis codes only. The net benefit of all three markers is reduced when only symptomatic codes are included. However, although the total number of cases is halved the APRI still detects over half of cases and the total number of referrals and unnecessary referrals per 100,000 is greatly reduced.

Tables S17 and S18 and Fig. S5 illustrate the effect of varying the length of the exclusion period. These show that although the overall number of referrals and net benefit values reduce slightly even when the exclusion period is extended from 30 to 180 days, the overall picture that the APRI is superior to the FIB‐4 at lower risk thresholds still holds.

### Comparison with the excluded patients

Table S19 compares patient characteristics between the analysis cohort and the subjects excluded due to not having a FIB‐4. The relative proportions in each category are very similar, although the analysis cohort seems to have a slightly higher risk of cirrhosis/HCC. This is echoed by the rates and cumulative incidence in Table S20.

When compared to the overall incidence rate ratio in the main cohort in Table S7, the ratio of the analysis cohort to the excluded subjects is around 1.51 (1.10/0.73) with the 10‐year risk ratio around 1.40 (0.95/0.68). This suggests there are three cases in the analysis cohort to every two cases in the excluded subjects, per 1000 person years.

## Discussion

We found the utility in the Welsh general population of three non‐invasive liver fibrosis markers varied substantially depending on the group targeted for testing. If we consider a low risk threshold (3%, comparable to National Institute for Health and Care Excellence [NICE] guidance on early detection of cancer) [33] then the APRI correctly predicted 71% of cases, performing better than FIB‐4 and AST/ALT. However, this approach would result in significant resource implications and, potentially, patient anxiety with nearly 90% of referrals being ‘unnecessary’, that is, false positive. In contrast, a more targeted case finding approach in populations with risk factors increases the proportion of true positive identifications and reduces the absolute numbers needed to be seen within the health service.

Implementation of the case‐finding modelled here is dependent on many factors; the availability of blood test results, the thresholds for referral chosen, the context in which such thresholds are applied, and the capacity of the system. For example, there may be a drive to reduce liver disease in certain risk groups, such as those with diabetes and obesity, not simply because they are at increased risk, but because they are more routinely tested already or represent a greater burden on the health service in the long run if the disease is left unchecked. Furthermore, the benefits of early diagnosis must be weighed against the consequences of investigating ‘false positive’ results, as this can lead to unnecessary invasive procedures for some (such as biopsy) which have their own associated risks.

The original studies in which the liver markers were developed were very small, specific populations, and we have shown their cut‐points for significant fibrosis/cirrhosis rarely align with actual observed risks in the general population [11, 12, 13]. For example, the APRI cut‐point of 1.50, originally developed to identify significant fibrosis in subjects with chronic hepatitis C, is much higher than all the cut‐points we identified, even at a 10‐year risk level for cirrhosis of 10%. In contrast, the approach of our study utilized a DCA method devised by Vickers [10], called net benefit, which can be compared across a range of thresholds and includes the trade‐off between true and false positives. In addition, our method did not assume a linear relationship between markers and liver disease risk or impose predefined categories, which is a superior approach to traditional methods of using binary measures of diagnostic accuracy [29]. Implementing DCA in different risk subgroups demonstrated that testing high risk groups could potentially reduce the overall number of referrals and simultaneously detect a higher proportion of cases within each subgroup. However, this still results in a significant number of false negative cases (i.e., people who will develop cirrhosis) being missed in the general population.

Our study has focussed on a prognostic yield of 3% for each marker, comparable with NICE guidelines on early detection of cancer. However, those guidelines are focussed on diagnosis not prognosis, so there may be a more suitable threshold level in this scenario. In addition, selection of the risk threshold to enact should be guided by a clinical consideration of the relative benefits and harms at each threshold, with the best strategy (in our case, fibrosis marker) being chosen that maximizes the net benefit. For example, a risk threshold of 3% implies the harm of 29 unnecessary referrals is roughly equal to the benefit of one correctly identified case. A risk threshold of 20% implies the harm of around five unnecessary referrals is equal to the benefit of one correctly identified case. The marker that performs best in these two examples would be the APRI and FIB‐4, respectively, but the initial selection of threshold (or range of thresholds) should ideally come first before the final strategy is identified.

Therefore, we assessed different risk thresholds using opportunistically assessed blood test markers in the early detection of liver disease. Our comparison across a range of thresholds and risk populations allows the selection of a marker (and corresponding cutoff) at the threshold deemed most useful from a clinical sense to best balance the trade‐off between identifying cases for investigation and referring patients who would not go on to develop liver disease. Our analyses at three different thresholds of 1%, 3% and 10% are not intended as a means of selecting the optimal risk threshold but are merely presented as examples to reflect the proportion of cases that would be correctly identified and the burden on the health service in terms of referrals should any of the three fibrosis markers be implemented at each of those thresholds.

For example, Table S10 shows a possible strategy utilizing a 1% risk level for the APRI trebles the number of referrals in the main cohort compared to a corresponding 3% risk, which despite identifying a further 9% of cases would likely overwhelm secondary care. In contrast, using the APRI for a 1% risk level in the group with obesity and diabetes, the number of referrals only doubles compared to the 3% risk level, but with the same number of cases detected. Table S11 shows that using a risk level of 10% decreases referrals in all groups, but at the cost of reducing the proportion of true cases detected.

Our study is the first we know of that has evaluated these markers at different risk thresholds to those traditionally utilized. We have analyzed all available primary care liver blood test data in SAIL which includes up to 79% of Welsh GP patients who are representative of the Welsh population as a whole [17]. Previous studies using traditional measures of diagnostic performance such as the area under the curve (AUC) and binary exposures are potentially problematic because they do not address clinical utility in the same way DCA does. For example, two recent, relatively small studies concluded non‐invasive markers were more suited to screening higher risk groups rather than the general population [34, 35]. A very large study (*n* = 537,250) that included 35% of inhabitants of Stockholm County concluded the FIB‐4 and AST/ALT were poor predictors of future alcohol‐related liver disease [36]. Another very large study (*n* = 416,200) that developed a risk score, LiverRisk, for predicting liver‐related mortality and HCC concluded it had superior performance compared to the FIB‐4 and APRI [37]. However, none of these studies examined the performance of the markers over a range of thresholds using DCA as we have.

As we started our study, another risk score, termed CORE, has been published [38]. This Swedish study was developed in a very large healthy cohort of almost half a million and further validated in two large cohorts from Finland and the United Kingdom. However, it was not validated in an unselected general population cohort as in our study. The score includes age, sex, ALT, AST and gamma‐glutamyl transferase (GGT) and was shown via several methods, including DCA, to be superior to the FIB‐4 at estimating 10‐year risk of cirrhosis. Although they did not quantify their results in the same way we have done, with numbers of referrals at different thresholds, and their method by its very nature includes an additional level of complexity, it may be that a new model such as this is needed in order to make the best use of routinely collected blood test data for predicting 10‐year risk of cirrhosis.

Other cohort studies are worth comparing to ours in more general terms. Two recent Swedish studies utilizing a general population cohort found a 10‐year cumulative incidence of 18% and 7.3% for the APRI and FIB‐4, respectively [39, 40]. Another study involving subjects with Type II diabetes and/or obesity found a 10‐year cumulative incidence of 15% for the FIB‐4 [41]. A UK Biobank study, restricted to subjects with risk factors for chronic liver disease, found a 10‐year cumulative incidence of 14.8% for the APRI at the 99th percentile [42]. All three studies used similar composite outcomes of cirrhosis/HCC that we employed. Although the two Swedish studies utilized a high cut‐point of 2.67 on the FIB‐4, rather than 3.25, and the prevalence of liver disease in the third study was slightly lower than ours, at around 0.6%, their results are not too dissimilar to ours shown in Table S7.

Our final cohort included the full spectrum of liver diseases with all possible aetiologies. Hence, the results are applicable to case‐finding for advanced fibrosis associated with any of these disease types but cannot be extrapolated to a single aetiology. Furthermore, our results have focussed on a UK context, with populations based on established UK risk factors. Although there will be locations with similar patient demographics, risk factors and health care systems, there will also be many populations in which these similarities do not exist. In countries where there is little alcohol consumption or lower rates of diabetes and obesity, the underlying rate of cirrhosis in the population may be much lower and require a different approach. Conversely, there may be locations where cirrhosis is more prevalent, or due to other causes such as viral hepatitis which require recalibration of the marker scores needed to identify subjects at certain risk levels. In addition, not all locations may have health services that provide for routine blood testing or the same capacity to deal with referrals that the United Kingdom allows.

Additionally, our final cohort are slightly more at risk of cirrhosis/HCC than the general population it is drawn from, as demonstrated by the numbers in Tables S7 and S20. This is to be expected as the analysis cohort represents those deemed to be at risk of liver disease by their managing clinician, hence the measurement of liver‐related blood tests. This means the range of suitable risk thresholds we have identified from our analysis may not be completely applicable to the general population. This statement is supported by the analysis restricted to symptomatic outcome codes, presented in Table S16 and Fig. S4, which show our cohort may be partly composed of subjects more likely to be tested for the outcome.

There has not been a validation study of the coding of cirrhosis within the SAIL databank. Although this is a limitation of our study, we have combined case definitions from primary and secondary care in a comparable way to similar routine database studies in the United Kingdom, which have shown reliable estimates of cirrhosis rates [43].

There are challenges involved in the implementation of the method we present here. First, the selection bias we describe above, where our study participants are more likely to have liver disease than a true general population, would likely cause our estimates of marker cut‐points to be slightly lower and estimates of the number of subjects with each risk factor to be higher than they actually are. Consequently, we may have underestimated the number of referrals and overestimated the number of true positive cases at each risk level.

Second, utilizing any of the markers we investigated would require reintroduction of the AST into routine care in the United Kingdom, which might encounter resistance by the system that has gradually phased this out over the last decade or so [18]. This has possibly been due to the notion that because the AST is less liver specific than other markers, it has little value as a general blood test and is an unnecessary expense. Clinicians may also need convincing the additional testing is justified and represents good value from a patient care perspective. Lastly, the patients themselves may not buy into the idea they are at increased risk of liver disease over a 10‐year period and may have to change their habits, just because of a single blood test result.

Through our analysis, we have demonstrated it is possible to utilize routine biomarkers to predict cirrhosis/HCC up to 10 years later. However, applying the markers at any threshold in the general population results in a large number of ‘unnecessary’ referrals, which makes them difficult to recommend, unless limited to higher risk groups. Finally, with the current worldwide increase in liver disease, we believe investigating the performance of cheap, easily available markers for either population screening or targeted case‐finding is more relevant than ever.

## Conclusions

Commonly utilized liver fibrosis markers, APRI, FIB‐4 and AST/ALT, are associated with a wide variation of referral thresholds for advanced fibrosis/cirrhosis and, depending on which group they are applied to, result in very different performance characteristics. DCA has shown the APRI has superior performance to FIB‐4 and AST/ALT at risk thresholds that might be suited to targeted case‐finding in subgroups if linked to further non‐invasive testing with Fibroscan or similar.

However, implementation of any case‐finding approach depends on balancing several factors. These include, but are not limited to, the costs, feasibility and capacity of the health service to deal with increased testing and referrals, re‐instatement of the AST as a routine element of blood testing, a clinical judgement on the relative importance of capturing cirrhosis cases versus ‘unnecessary’ referrals, and assessment of quality adjusted life years (QALYs) of any treatment options.

## Author contributions

All authors contributed to the design of the study, analysis and interpretation of the data, the drafting of the article and the final agreed version.

## Conflict of interest statement

The authors declare no conflicts of interest.

## Funding information

TAH salary is 50% funded by the NIHR Nottingham Biomedical Research Centre (BRC). In addition, all authors are affiliated with the NIHR Nottingham BRC. For the purpose of Open Access, the author has applied a Creative Commons Attribution (CC BY) licence to any Author Accepted Manuscript version arising.

## Ethics statement

This study was approved by the SAIL Information Governance Review Panel (IGRP), reference 0842.

## Supporting information




**Table S1** Liver function test Read codes included in the data, by type.
**Table S2**: Final cirrhosis code list, matched to consensus list produced by Shearer et al. (2022).
**Table S3**: Read v2 codes used to define diabetes.
**Table S4**: Read v2 codes used to define hazardous alcohol use.
**Table S5**: Read v2 codes used to define BMI and obesity.
**Table S6**: Model checking results for the main cohort and individual subgroups.
**Table S7**: Incidence rates by demographic characteristic, for patients with all three markers.
**Table S8**: Final fractional polynomial models suggested by the MFP routine for the main analyses.
**Table S9**: Net benefit and diagnostic results for all three markers at several thresholds, for predicting cirrhosis/HCC within 10 years of first exposure.
**Table S10**: Possible case‐finding strategies for a 1% risk of cirrhosis/HCC.
**Table S11**: Possible case‐finding strategies for a 10% risk of cirrhosis/HCC.
**Table S12**: Calculation of size of each pseudo‐population.
**Table S13**: Possible case‐finding strategies for a 3% risk of cirrhosis/HCC, cause‐specific analysis (does not take account of competing risk of death).
**Table S14**: Equivalent marker values associated with each threshold, by age group.
**Table S15**: Harrell's concordance C‐statistic for each fibrosis marker, for different periods of follow‐up time.
**Table S16**: Comparing number of referrals at the 3% risk threshold for the main analysis with symptomatic codes only*.
**Table S17**: Numbers of cirrhosis/HCC cases remaining for analysis after applying different exclusion periods.
**Table S18**: Comparison of numbers of referrals per 100,000 for 30‐ and 180‐day exclusion periods.
**Table S19**: Demographic characteristics of analysis cohort compared with excluded subjects.
**Table S20**: Incidence rates by demographic characteristic, for patients without a FIB‐4 omitted from the main analysis.
**Fig. S1**: Study design diagram.
**Fig. S2**: Log‐hazard ratio with 95% confidence region versus each fibrosis marker score.
**Fig. S3**: DCA plots comparing net benefit in subjects aged <65 years (left) with those aged > = 65 years (right).
**Fig. S4**: Comparing the DCA curve for the outcome restricted to symptomatic cirrhosis codes (left) with the original analysis including all cirrhosis codes (right).
**Fig. S5**: DCA curve comparing net benefit for a 180‐day exclusion period (left) with the original 30‐day exclusion period (right).

## Data Availability

Data may be obtained from a third party and are not publicly available. The data used in this study are available in the SAIL databank at Swansea University, Swansea, UK. All proposals to use SAIL data are subject to review by an independent Information Governance Review Panel (IGRP). Before any data can be accessed, approval must be given by the IGRP. The IGRP gives careful consideration to each project to ensure proper and appropriate use of SAIL data. When access has been granted, it is gained through a privacy‐protecting safe haven and remote access system referred to as the SAIL Gateway. SAIL has established an application process to be followed by anyone who would like to access data via SAIL https://www.saildatabank.com/application‐process.
