# Supplementary material for: Identifying subjects at risk of liver cirrhosis via a range of thresholds for common fibrosis markers: A Welsh general population‐based cohort study
Source: J Intern Med. 2026 Jan 7;299(3):398–413. doi: 10.1111/joim.70064 (PMC12868999; doi:10.1111/joim.70064)
Supplement: Supplementary file 1 — Table S1 Liver function test Read codes included in the data, by type. Table S2: Final cirrhosis code list, matched to consensus list produced by Shearer et al. (2022). Table S3: Read v2 codes used to define diabetes. Table S4: Read v2 codes used to define hazardous alcohol use. Table S5: Read v2 codes used to define BMI and obesity. Table S6: Model checking results for the main cohort and individual subgroups. Table S7: Incidence rates by demographic characteristic, for patients with all three markers. Table S8: Final fractional polynomial models suggested by the MFP routine for the main analyses. Table S9: Net benefit and diagnostic results for all three markers at several thresholds, for predicting cirrhosis/HCC within 10 years of first exposure. Table S10: Possible case‐finding strategies for a 1% risk of cirrhosis/HCC. Table S11: Possible case‐finding strategies for a 10% risk of cirrhosis/HCC. Table S12: Calculation of size of each pseudo‐population. Table S13: Possible case‐finding strategies for a 3% risk of cirrhosis/HCC, cause‐specific analysis (does not take account of competing risk of death). Table S14: Equivalent marker values associated with each threshold, by age group. Table S15: Harrell's concordance C‐statistic for each fibrosis marker, for different periods of follow‐up time. Table S16: Comparing number of referrals at the 3% risk threshold for the main analysis with symptomatic codes only*. Table S17: Numbers of cirrhosis/HCC cases remaining for analysis after applying different exclusion periods. Table S18: Comparison of numbers of referrals per 100,000 for 30‐ and 180‐day exclusion periods. Table S19: Demographic characteristics of analysis cohort compared with excluded subjects. Table S20: Incidence rates by demographic characteristic, for patients without a FIB‐4 omitted from the main analysis. Fig. S1: Study design diagram. Fig. S2: Log‐hazard ratio with 95% confidence region versus each fibrosis marker score. Fig. S3: DCA plots comparing n [file JOIM-299-398-s001.docx]

**SUPPLEMENTARY MATERIAL**

**TITLE:** Identifying subjects at risk of liver cirrhosis via a range of thresholds for common fibrosis markers: a Welsh general population-based cohort study

Mr Trevor A. Hill^1, 4^ (Corresponding author)

Professor Joe West^2, 3, 4^

Professor Joanne R. Morling^2, 4^

Dr Colin J. Crooks^1, 4^

^1^ Translational Medical Sciences, School of Medicine, University of Nottingham, UK

^2^ Lifespan and Population Health, School of Medicine, University of Nottingham, UK

^3^ Department of Clinical Medicine, Aarhus University, Aarhus, Denmark

^4^ Gastrointestinal and Liver Theme, National Institute for Health Research (NIHR) Nottingham Biomedical Research Centre (BRC), Nottingham University Hospitals NHS Trust and the University of Nottingham, School of Medicine, Queen’s Medical Centre, Nottingham, NG7 2UH, United Kingdom

**Table of contents**

**CODE LISTS:**

| Table S1: Liver function test Read codes included in the data, by type | 3 |
| --- | --- |
| Table S2: Final list of cirrhosis codes | 4 |
| Table S3: Read v2 codes used to define diabetes | 7 |
| Table S4: Read v2 codes used to define hazardous alcohol use | 8 |
| Table S5: Read v2 codes used to define BMI and obesity | 12 |

**MODEL CHECKING AND STUDY DETAILS:**

| Table S6: Model checking results for the main cohort and individual sub-groups | 13 |
| --- | --- |
| Table S8: Final fractional polynomial models suggested by the MFP routine for the main analyses | 17 |
| Table S12: Calculation of size of each pseudo-population | 22 |
| Figure S1: Study design diagram | 29 |
| Figure S2: Log-hazard ratio with 95% confidence region vs. each fibrosis marker score | 30 |

**Table of contents continued**

**INCIDENCE RATES:**

| Table S7: Incidence rates by demographic characteristic, for patients with all three markers | 15 |
| --- | --- |
| Table S20: Incidence rates by demographic characteristic, for patients without a FIB-4 omitted from the main analysis | 28 |

**NET BENEFIT AND FURTHER MAIN RESULTS:**

| Table S9: Net benefit and diagnostic results for all three markers at several thresholds, for predicting cirrhosis/HCC within 10 years of first exposure. | 18 |
| --- | --- |
| Table S10: Possible case-finding strategies for a 1% risk of cirrhosis/HCC | 20 |
| Table S11: Possible case-finding strategies for a 10% risk of cirrhosis/HCC | 21 |

**SENSITIVITY ANALYSES*:**

| Table S13: Possible case-finding strategies for a 3% risk of cirrhosis/HCC, cause-specific analysis (does not take account of competing risk of death) | 23 |
| --- | --- |
| Table S14: Equivalent marker values associated with each threshold, by age group | 24 |
| Table S15: Harrell’s concordance C-statistic for each fibrosis marker, for different periods of follow-up time | 25 |
| Table S16: Comparing number of referrals at the 3% risk threshold for the main analysis with symptomatic codes only | 25 |
| Table S17: Numbers of cirrhosis/HCC cases remaining for analysis after applying different exclusion periods | 26 |
| Table S18: Comparison of numbers of referrals per 100,000 for 30-day and 180-day exclusion periods | 26 |
| Table S19: Demographic characteristics of analysis cohort compared with previous samples | 27 |
| Figure S3: DCA plots comparing net benefit in subjects aged <65 years (left) with those aged >=65 years (right) | 31 |
| Figure S4: Comparing the DCA curve for the outcome restricted to symptomatic cirrhosis codes (left) with the original analysis including all cirrhosis codes (right). | 32 |
| Figure S5: DCA curve comparing net benefit for a 180-day exclusion period (left) with the original 30-day exclusion period (right). | 33 |

* Sensitivity analyses are cause-specific, cirrhosis/HCC only, and do not take account of competing risk of death.

**Table S1: Liver function test Read codes included in the data, by type**

| **Blood test type** | **Read code and description** |
| --- | --- |
| Platelet count | 42P.. Platelet count  42P1. Platelet count normal  42P4. Platelet count abnormal  42PZ. Platelet count NOS |
| ALT | 44G3. ALT/SGPT serum level  44G30 ALT/SGPT level normal  44G31 ALT/SGPT level abnormal  44GA. Plasma alanine aminotransferase level  44GB. Serum alanine aminotransferase level  44S8. Serum alanine level |
| AST | 44H5. AST – aspartate transam. (SGOT)  44H50 AST/SGOT level normal  44H51 AST/SGOT level abnormal  44H52 AST/SGOT level raised  44HB. AST serum level  44HC. Plasma aspartate transaminase level |

**Table S2: Final cirrhosis code list, matched to consensus list produced by Shearer et al 2022.**

| **Shearer ICD10 consensus code list** | **Matched ICD10, Read v2 and OPCS4 codes included in our analysis** |
| --- | --- |
| K74.6 Other and unspecified cirrhosis of the liver | **ICD10 codes from the PEDW:**  K74.6 Other and unspecified cirrhosis of the liver  **Read codes from the GP event data:**  J61.. Cirrhosis and chronic liver disease  J615. Cirrhosis – non-alcoholic  J6153 Diffuse nodular cirrhosis  J615H Infectious cirrhosis NOS  J615z Non-alcoholic cirrhosis NOS  Jyu71 [X]Other and unspecified cirrhosis of liver |
| K70.3 Alcoholic cirrhosis of the liver | **ICD10 codes from the PEDW:**  K70.3 Alcoholic cirrhosis of liver, unspecified  **Read codes from the GP event data:**  J612. Alcoholic cirrhosis of liver |
| I85 Oesophageal varices  I85.0 With bleeding  I85.9 Without bleeding | **ICD10 codes from the PEDW:**  I85.0 OV with bleeding  I85.9 OV without bleeding  **Read codes from the GP event data:**  G8522 Oesophageal varices in cirrhosis of the liver  G8523 Oesophageal varices in alcoholic cirrhosis of the liver  G858. Oesophageal varices NOS  G850. Oesophageal varices with bleeding  G851. Oesophageal varices without bleeding  **Further Read codes from GP event data:**  7609. Open operations on oesophageal varices  76093 Local ligation of oesophageal varices  76094 Open injection sclerotherapy to oesophageal varices  7609y Other specified open operation on oesophageal varices  7609z Open operation on oesophageal varices NOS  760C3 Fibreoptic endoscopic injection sclerotherapy oesoph varices  760C5 Fibreoptic endoscopic banding of oesophageal varices  760F3 Rigid oesophagoscopic injection sclerotherapy oesoph varices  760F4 Rigid oesophagoscopic banding of oesophageal varices  761D8 Fibreoptic endoscopic rubber band ligation of upper GIT varices  **OPCS4 codes from PEDW data:**  G10.4 Local ligation of varices of oesophagus  G10.5 Open injection sclerotherapy to varices of oesophagus  G10.8 Other specified open operations on varices of oesophagus  G10.9 Unspecified open operations on varices of oesophagus  G14.4 Fibreoptic endoscopic injection sclerotherapy to varices of oesophagus  G17.4 Endoscopic injection sclerotherapy to varices of oesophagus using rigid oesophagoscope  G43.7 Fibreoptic endoscopic rubber band ligation of upper gastrointestinal tract varices |
| I98 Oesophageal varices in diseases classified  elsewhere  I98.2 without bleeding  I98.3 with bleeding | **ICD10 codes from the PEDW:**  I98.2 OV in diseases classified elsewhere  I98.3 OV with bleeding in diseases classified elsewhere  **Read codes from the GP event data:**  G852. Oesophageal varices in diseases EC  G852z Oesophageal varices in diseases EC NOS  G8521 Oesophageal varices without bleeding in diseases EC  G8520 Oesophageal varices with bleeding in diseases EC |
| K76.6 Portal hypertension | **ICD10 codes from the PEDW:**  K76.6 Portal hypertension  **Read codes from the GP event data:**  J623. Portal hypertension  J6151 Multilobular portal cirrhosis  J6154 Fatty portal cirrhosis  J6155 Hypertrophic portal cirrhosis  J6156 Capsular portal cirrhosis  J6158 Juvenile portal cirrhosis  J615G Zooparasitic portal cirrhosis  J615y Portal cirrhosis unspecified  **OPCS4 codes from PEDW data:**  J06.1 Transjugular intrahepatic insertion of stent into portal vein  J06.2 Transjugular intrahepatic insertion of stent graft into portal vein |
| K72.9 Hepatic failure, unspecified | **ICD10 codes from the PEDW:**  K72.1 Chronic hepatic failure*  K72.9 Hepatic failure, unspecified |
| K76.7 Hepatorenal syndrome | **ICD10 codes from the PEDW:**  K76.7 Hepatorenal syndrome |
| C22.0 Liver cell carcinoma* | **ICD10 codes from the Welsh cancer registry:**  C22.0 Liver cell carcinoma  **ICD9 codes from the Welsh cancer registry:**  1550 Malignant neoplasm of liver (& morphology==81703)  **NB: the ICD9 code 1550 is only included if the morphology code is also coded 81703.** |

* Additional codes, not included in Shearer’s consensus list.

N= 55 codes in total (n=11 ICD10 codes (10 + 1 liver cell carcinoma code), n=1 ICD9 code, n=9 OPCS4 codes, n=34 Read codes)

**Table S3: Read v2 codes used to define diabetes**

| **Diabetes code and description** | **Male** | **Female** | **All subjects** |
| --- | --- | --- | --- |
| C10F. Type 2 diabetes mellitus | 5,453 (56.0%) | 4,282 (53.3%) | 9,735 (54.7%) |
| C10.. Diabetes mellitus | 2,251 (23.1%) | 2,001 (24.9%) | 4,252 (23.9%) |
| C109. Non-insulin dependent diabetes mellitus | 972 (10.0%) | 837 (10.4%) | 1,809 (10.2%) |
| C10E. Type 1 diabetes mellitus | 420 (4.3%) | 382 (4.8%) | 802 (4.5%) |
| C1001 Diabetes mellitus, adult onset, no mention of complication | 314 (3.2%) | 272 (3.4%) | 586 (3.3%) |
| C108. Type 1 / insulin dependent diabetes mellitus | 116 (1.2%) | 98 (1.2%) | 214 (1.2%) |
| C1000 Insulin dependent diabetes mellitus | 60 (0.6%) | 38 (0.5%) | 98 (0.6%) |
| C10FJ Insulin treated type 2 diabetes mellitus | 49 (0.5%) | 39 (0.5%) | 88 (0.5%) |
| Other diabetes code | 110 (1.1%) | 90 (1.1%) | 200 (1.1%) |
| Total | 9,745 (54.8%) | 8,039 (45.2%) | 17,784 (100.0%) |

NB: Although all Read v2 codes beginning with ‘C10’ were extracted, this table only shows the codes used to define diabetes for the subjects included in the final analysis. Male and female percentages are row percentages, the percentage shown in the final column is a column percentage.

**Table S4: Read v2 codes used to define hazardous alcohol use**

| **Code** | **Description** |
| --- | --- |
| Main code list taken from Askgaard et al, 2021 | |
| J610.00 | Alcoholic fatty liver |
| J611.00 | Acute alcoholic hepatitis |
| J612000 | Alcoholic fibrosis and sclerosis of liver |
| J613.00 | Alcoholic liver damage unspecified |
| J613000 | Alcoholic hepatic failure |
| J617.00 | Alcoholic hepatitis |
| J617000 | Chronic alcoholic hepatitis |
|  |  |
| 13ZY.00 | Disqualified from driving due to excess alcohol |
| 1B1c.00 | Alcohol induced hallucinations |
| 8BA8.00 | Alcohol detoxification |
| C150500 | Alcohol-induced pseudo-Cushing's syndrome |
| F11x000 | Cerebral degeneration due to alcoholism |
| F25B.00 | Alcohol-induced epilepsy |
| F375.00 | Alcoholic polyneuropathy |
| J671000 | Alcohol-induced chronic pancreatitis |
| ZV11311 | [V]Problems related to lifestyle alcohol use |
|  |  |
| E01..00 | Alcoholic psychoses |
| E010.00 | Alcohol withdrawal delirium (including E010. Delirium tremens) |
| E011.00 | Alcohol amnestic syndrome |
| E011000 | Korsakov's alcoholic psychosis |
| E011100 | Korsakov's alcoholic psychosis with peripheral neuritis |
| E011z00 | Alcohol amnestic syndrome NOS |
| E012.00 | Other alcoholic dementia (including E012.11 Alcoholic dementia NOS) |
| E012000 | Chronic alcoholic brain syndrome |
| E013.00 | Alcohol withdrawal hallucinosis |
| E014.00 | Pathological alcohol intoxication |
| E015.00 | Alcoholic paranoia |
| E01y.00 | Other alcoholic psychosis |
| E01y000 | Alcohol withdrawal syndrome |
| E01yz00 | Other alcoholic psychosis NOS |
| E01z.00 | Alcoholic psychosis NOS |
| E0112* | Wernicke-Korsakov syndrome |
|  |  |
| E23..00 | Alcohol dependence syndrome (including E23..11 Alcoholism) |
| E230.00 | Acute alcoholic intoxication in alcoholism |
| E230000 | Acute alcoholic intoxication, unspecified, in alcoholism |
| E230100 | Continuous acute alcoholic intoxication in alcoholism |
| E230200 | Episodic acute alcoholic intoxication in alcoholism |
| E230z00 | Acute alcoholic intoxication in alcoholism NOS |
| E2303* | Acute alcoholic intoxication in remission, in alcoholism |
| E231.00 | Chronic alcoholism |
| E231000 | Unspecified chronic alcoholism |
| E231100 | Continuous chronic alcoholism |
| E231200 | Episodic chronic alcoholism |
| E231z00 | Chronic alcoholism NOS |
| E23z.00 | Alcohol dependence syndrome NOS |
| E2313* | Chronic alcoholism in remission |
|  |  |
| E250.00 | Nondependent alcohol abuse  (including E250.12 Hangover and E250.14 Intoxication) |
| E250000 | Nondependent alcohol abuse, unspecified |
| E250100 | Nondependent alcohol abuse, continuous |
| E250200 | Nondependent alcohol abuse, episodic |
| E250z00 | Nondependent alcohol abuse NOS |
| E2503* | Nondependent alcohol abuse in remission |
|  |  |
| Eu10.00 | [X]Mental and behavioural disorders due to use of alcohol |
| Eu10000 | [X]Mental & behav dis due to use alcohol: acute intoxication  (including Eu100.11 Acute drunkenness) |
| Eu10100 | [X]Mental and behav dis due to use of alcohol: harmful use |
| Eu10200 | [X]Mental and behav dis due to use alcohol: dependence syndr  (including Eu10211 and Eu10212) |
| Eu10300 | [X]Mental and behav dis due to use alcohol: withdrawal state |
| Eu10411 | [X]Delirium tremens, alcohol induced |
| Eu10500 | [X]Mental & behav dis due to use alcohol: psychotic disorder  (including Eu10511, Eu10512, Eu10513) |
| Eu10600 | [X]Mental and behav dis due to use alcohol: amnesic syndrome (including Eu10611) |
| Eu10711 | [X]Alcoholic dementia NOS (including Eu10712) |
| Eu10800 | [X]Alcohol withdrawal-induced seizure |
| Eu10y00 | [X]Men & behav dis due to use alcohol: oth men & behav dis |
| Eu10z00 | [X]Ment & behav dis due use alcohol: unsp ment & behav dis |
|  |  |
| SM0..00 | Alcohol causing toxic effect |
| SM00.00 | Ethyl alcohol causing toxic effect |
| SM00000 | Ethanol causing toxic effect |
| SM00100 | Denatured alcohol causing toxic effect |
| SM00z00 | Ethyl alcohol causing toxic effect NOS |
| SM0y.00 | Other alcohol causing toxic effect |
| SM0z.00 | Alcohol causing toxic effect NOS |
|  |  |
| Additional codes* | |
| 136..00 | Alcohol consumption |
| 136V.00 | Alcohol units per week |
| 136X.00 | Alcohol units consumed on heaviest drinking day |
| 38Dz.00 | Severity of alcohol dependence questionnaire |
|  |  |
| 1365. | Heavy drinker - 7-9u/day |
| 1366. | Very heavy drinker - greater than 9 units/day |
| 136D. | Ex-heavy drinker - (7-9u/day) |
| 136E. | Ex-very heavy drinker - greater than 9 units/day |
| 136K. | Alcohol intake above recommended sensible limits |
| 136a.00 | Increasing risk drinking |
| 136c.00 | Higher risk drinking |
| 136P.00 | Heavy drinker |
| 136Q.00 | Very heavy drinker |
| 136R.00 | Binge drinker |
| 136S.00 | Hazardous alcohol use |
| 136T.00 | Harmful alcohol use |
| 136W.00 | Alcohol misuse |
| 136Y.00 | Drinks in morning to get rid of hangover |
|  |  |
| 13Y8. | Alcoholics anonymous |
| 1462. | H/O: alcoholism |
| 66e..00 | Alcohol disorder monitoring |
| 66e0.00 | alcohol abuse monitoring |
| 7P22100 | Delivery of rehabilitation for alcohol addiction |
| 8CAM000 | Advised to abstain from alcohol consumption |
| 8CAv.00 | advised to contact primary care alcohol worker |
| 8G32. | Aversion therapy - alcoholism |
| 8H35. | Admitted to alcohol detoxification centre |
| 8H7p.00 | Referral to community alcohol team |
| 8HHe.00 | Referral to community drug and alcohol team |
| 8HkG.00 | Referral to specialist alcohol treatment service |
| 8HkJ.00 | Referral to alcohol brief intervention service |
| 8IAF.00 | brief intervention for excessive alcohol consumptn declined |
| 8IAJ.00 | declined referral to specialist alcohol treatment service |
| 8IAt.00 | Extended intervention for excessive alcohol consumption declined |
|  |  |
| 9k1..00 | Alcohol misuse - enhanced services administration |
| 9k11.00 | Alcohol consumption counselling |
| 9k12.00 | Alcohol misuse - enhanced service completed |
| 9k19.00 | Alcohol assesment declined - enhanced services admin |
| 9k1A.00 | Brief intervention for excessive alcohol consumptn completed |
| 9k1B.00 | Extended intervention for excessive alcohol consumption completed |
| 9NN2.00 | Under care of community alcohol team |
| SLH3.00 | alcohol deterrent poisoning |
|  |  |
| C251. | Thiamine (vit B1) defic state: [Wernicke] or [oth & unspec] |
| F1440 | Alcoholic cerebellar degeneration |
| F394100 | Alcoholic myopathy |
| G555. | Alcohol-induced heart muscle disease |
| J153. | Alcoholic gastritis |
| R103. | [D]Alcohol blood level excessive |
|  |  |
| U804. | [X]Eviden of alcohl involv blood alcohl level 80-99mg/100ml |
| U805. | [X]Eviden of alcoh involv blood alcoh level 100-119mg/100ml |
| U806. | [X]Eviden of alcoh involv blood alcoh level 120-199mg/100ml |
| U807. | [X]Eviden of alcoh involv blood alcoh level 200-239mg/100ml |
| U808. | [X]Eviden alcoh involv blood alcoh level 240mg/100ml or more |
|  |  |
| Z191.00 | alcohol detoxification |
| Z191100 | alcohol withdrawal regime |
| Z191200 | Planned reduction of alcohol consumption  (including Z191211 alcohol reduction programme) |
| Z191400 | Self-monitoring of alcohol intake |
| Z4B1.00 | Alcoholism counselling |
| ZC22200 | Advice to change alcoholic drink intake |
| ZC2H.00 | Advice to change alcohol intake |
| ZG23100 | Advice on alcohol consumption |
| ZV57A00 | [V]Alcohol rehabilitation |
| ZV6D6 | [V]Alcohol abuse counselling and surveillance |

NB: This list represents the codes extracted from the main SAIL cohort. Once merged with the analysis cohort of patients with all three fibrosis markers, not all codes were represented.

* Codes marked with an asterisk were not included in the original list by Askgaard et al and have been added from other sources.

**Table S5: Read v2 codes used to define BMI and obesity**

| **Code** | **Description** |
| --- | --- |
| 222A. | O/E – obese |
| 22K.. | Body Mass Index |
| 22K1. | Body Mass Index normal K/M2 |
| 22K2. | Body Mass Index high K/M2 |
| 22K3. | Body Mass Index low K/M2 |
| 22K4. | Body Mass Index 25-29 – overweight |
| 22K5. | Body Mass Index 30+ – obesity |
| 22K6. | Body Mass Index less than 20 |
| 22K7. | Body Mass Index 40+ – severely obese |
| 22K8. | Body Mass Index 20-24 – normal |
|  |  |
| 22A.. | O/E – weight |
| 229.. | O/E – height |
| 229Z. | O/E – height NOS |
| 22A4. | O/E – weight 10-20% over ideal |
| 22A5. | O/E – weight >20% over ideal |
|  |  |
| 66C1. | Initial obesity assessment |
| 66C2. | Follow-up obesity assessment |
| 66C4. | Has seen dietician – obesity |
| 66C6. | Treatment of obesity started |
| 66CE. | Reason for obesity therapy – occupational |
|  |  |
| C380. | Obesity |
| C3800 | Obesity due to excess calories |
| C3803 | Morbid obesity |
| C3804 | Central obesity |
| C3805 | Generalised obesity |
|  |  |
| C38y0 | Pickwickian syndrome |
| C38z0 | Simple obesity NOS |
| Cyu70 | [X]Other obesity |
| ZC2CM | Dietary advice for obesity |

Note that this is not an exhaustive list of all BMI Read v2 codes initially extracted from the Welsh data. For example, there are other codes, such as *66C5. Treatment of obesity changed*, that although a valid code, were not matched to any patient in our final cohort. Only codes present in the final analysis cohort are presented here.

**Table S6: Model checking results for the main cohort and individual sub-groups**

|  | **FIB-4** | | | | **APRI** | | | | **AST/ALT** | | | |
| --- | --- | --- | --- | --- | --- | --- | --- | --- | --- | --- | --- | --- |
|  | PH test* | | | DFBETA** | PH test | | | DFBETA | PH test | | | DFBETA |
|  | rho | Chi2 | P>Chi2 | Number (%) | rho | Chi2 | P>Chi2 | Number (%) | rho | Chi2 | P>Chi2 | Number (%) |
| Main cohort  Term 1***  Term 2  Global test | -0.04524  0.01214  - | 7.32  0.58  74.56 | 0.0068  0.4476  0.0000 | 2,044 (1.0%)  1,976 (1.0%)  - | -0.03874  -0.00341  - | 3.37  0.03  46.85 | 0.0666  0.8739  0.0000 | 2,383 (1.2%)  2,123 (1.0%)  - | 0.11464  0.13130  - | 26.51  34.37  84.23 | 0.0000  0.0000  0.0000 | 1,705 (0.8%)  1,766 (0.9%)  - |
| Obese group  Term 1  Term 2  Global test | -0.01087  0.07934  - | 0.10  5.07  29.37 | 0.7569  0.0243  0.0000 | 449 (0.9%)  517 (1.1%)  - | -0.05593  -0.00878  - | 2.10  0.05  20.71 | 0.1469  0.8207  0.0000 | 578 (1.2%)  491 (1.0%)  - | -0.20454  0.07994  - | 21.58  3.99  32.02 | 0.0000  0.0458  0.0000 | 488 (1.0%)  400 (0.8%)  - |
| Diabetic group  Term 1  Term 2  Global test | -0.10656  0.07065  - | 4.11  1.92  10.75 | 0.0426  0.1657  0.0046 | 307 (1.7%)  289 (1.6%)  - | -0.06311  -0.00380  - | 1.40  0.01  6.61 | 0.2360  0.9403  0.0366 | 343 (1.9%)  269 (1.5%)  - | -0.20392  0.07549  - | 12.87  1.93  18.00 | 0.0003  0.1643  0.0001 | 258 (1.5%)  203 (1.1%)  - |
| Haz. alcohol  Term 1  Term 2  Global test | -0.05257  0.00962  - | 3.58  0.12  20.75 | 0.0586  0.7286  0.0000 | 620 (2.1%)  762 (2.6%)  - | -0.00998  -0.01911  - | 0.09  0.34  8.92 | 0.7604  0.5586  0.0115 | 835 (2.9%)  735 (2.5%)  - | -0.10018  0.04849  - | 8.57  2.07  31.33 | 0.0034  0.1501  0.0000 | 734 (2.5%)  668 (2.3%)  - |
| Obese and haz. alcohol  Term 1  Term 2  Global test | -0.18141  0.13921  - | 7.70  4.43  11.42 | 0.0055  0.0353  0.0033 | 178 (2.2%)  161 (2.0%)  - | -0.15053  0.10029  - | 4.89  2.24  8.68 | 0.0270  0.1348  0.0131 | 186 (2.3%)  162 (2.0%)  - | -0.13073  0.01241  - | 3.08  0.03  6.35 | 0.0790  0.8636  0.0418 | 171 (2.2%)  144 (1.8%)  - |
| Diabetic and obese  Term 1  Term 2  Global test | 0.07985  0.14315  - | 1.29  3.86  5.78 | 0.2564  0.0493  0.0555 | 126 (1.5%)  163 (1.9%)  - | -0.07393  0.04260  - | 0.95  0.33  2.62 | 0.3305  0.5681  0.2705 | 155 (1.8%)  144 (1.7%)  - | -0.24507  -  - | 8.67  -  8.67 | 0.0032  -  0.0032 | 136 (1.6%)  -  - |
| Diabetic and haz. alcohol  Term 1  Term 2  Global test | -0.16754  0.09333  - | 2.94  0.99  4.72 | 0.0865  0.3198  0.0945 | 87 (3.0%)  82 (2.9%)  - | -0.08920  0.01418  - | 0.74  0.02  2.36 | 0.3898  0.8902  0.3078 | 87 (3.0%)  75 (2.6%)  - | 0.23218  -  - | 5.68  -  5.68 | 0.0172  -  0.0172 | 77 (2.7%)  -  - |
| All three risk factors  Term 1  Term 2  Global test | 0.18565  -  - | 2.08  -  2.08 | 0.1495  -  - | 043 (3.0%)  -  - | -0.14253  0.11227  - | 1.21  0.77  1.69 | 0.2711  0.3790  0.4287 | 43 (3.0%)  44 (3.1%)  - | 0.08807  -  - | 0.39  -  0.39 | 0.5315  -  0.5315 | 42 (2.9%)  -  - |

* PH test via Schoenfeld residuals. ** Number and percentage of scaled DFBETAs outside the critical range defined by +/- (2/sqrt(N))

*** For example, Ifib4__1, Ifib4__2, Iapri_1, Iapri__2, Iasta__1 and Iasta__2. Some models include only one transformed term.

NB: Haz. alcohol = hazardous alcohol use.

**Table S7: Incidence rates by demographic characteristic, for patients with all three markers**

|  | **N patients** | **Person years** | **Failures**  **(cirrhosis/HCC)** | **Incidence rate of cirrhosis/HCC (per 1000 person-years)** | **Cumulative 10-year risk**  **/ incidence proportion (%)*** |
| --- | --- | --- | --- | --- | --- |
| All patients  Male  Female | 203,005 (100.0%)  88,269 (43.5%)  114,736 (56.5%) | 1,439,237.59  612,007.17  827,230.42 | 1,583  935  648 | 1.10 (1.05 – 1.16)  1.53 (1.43 – 1.63)  0.78 (0.73 – 0.85) | 0.95 (0.90 – 0.99)  1.30 (1.22 – 1.39)  0.68 (0.63 – 0.73) |
| Age when tested  18 – 39  40 – 59  60 – 79  80+ | 45,279 (22.30%)  69,095 (34.04%)  67,838 (33.42%)  20,793 (10.24%) | 327,363.48  534,272.55  481,885.90  95,715.66 | 150  685  643  105 | 0.46 (0.39 – 0.54)  1.28 (1.19 – 1.38)  1.33 (1.24 – 1.44)  1.10 (0.91 – 1.33) | 0.45 (0.38 – 0.53)  1.21 (1.12 – 1.30)  1.10 (1.02 – 1.19)  0.57 (0.47 – 0.69) |
| Deprivation quintile  Most deprived  Next most deprived  Median deprivation  Next least deprived  Least deprived  Missing | 33,722 (16.61%)  45,677 (22.50%)  47,273 (23.29%)  48,535 (23.91%)  24,848 (12.24%)  2,950 (1.45%) | 232,899.89  329,621.71  337,450.63  347,166.32  172,959.61  19,139.43 | 368  369  342  311  166  27 | 1.58 (1.43 – 1.75)  1.12 (1.01 – 1.24)  1.01 (0.91 – 1.13)  0.90 (0.80 – 1.00)  0.96 (0.82 – 1.12)  1.41 (0.97 – 2.06) | 1.34 (1.20 – 1.48)  0.97 (0.87 – 1.07)  0.88 (0.79 – 0.98)  0.77 (0.69 – 0.86)  0.83 (0.71 – 0.97)  1.13 (0.76 – 1.63) |
| High marker values  APRI High (>=1.5)  Low (<1.5)  FIB-4 High (>=3.25)  Low (<3.25)  AST/ALT High (>=1.0)  Low (<1.0)  AST/ALT High (>=2.0)  Low (<2.0) | 2,696 (1.33%)  200,309 (98.67%)  4,823 (2.38%)  198,182 (97.62%)  102,575 (50.53%)  100,430 (49.47%)  7,668 (3.78%)  195,337 (96.22%) | 10,399.13  1,428,838.46  19,048.09  1,420,189.50  716,557.00  722,680.59  41,247.72  1,397,989.87 | 388  1,195  539  1,044  1,066  517  297  1,286 | 37.31 (33.78 – 41.21)  0.84 (0.79 – 0.89)  28.30 (26.01 – 30.79)  0.74 (0.69 – 0.78)  1.49 (1.40 – 1.58)  0.72 (0.66 – 0.78)  7.20 (6.43 – 8.07)  0.92 (0.87 – 0.97) | 22.66 (20.58 – 24.80)  0.73 (0.69 – 0.78)  14.25 (13.13 – 15.42)  0.66 (0.62 – 0.70)  1.21 (1.14 – 1.28)  0.67 (0.61 – 0.73)  4.38 (3.91 – 4.89)  0.81 (0.77 – 0.86) |

NB: Incidence rate per 1000 person years is calculated as follows: [(number of failures) / (person years)] * 1000

e.g. for high APRI the incidence rate = (388/10399)*1000 = 37.31

* Calculated using the stcompet function in Stata, which provides a non-parametric estimate of the cumulative incidence by strata and does not utilise the fractional polynomial method.

**Table S8: Final fractional polynomial models suggested by the MFP routine for the main analyses**

| **Analysis/ marker** | **Final form of log-hazard ratios, showing coefficients, included in the Cox models** | **Where X=** |
| --- | --- | --- |
| Main analysis  FIB-4 Cirrhosis  Death  APRI Cirrhosis  Death  AST/ALT Cirrhosis  Death | ln(HR) = (51.52566 * (sqrt(X) - 0.108180083)) + (-66.59444 * (X - 0.0117029303))  ln(HR) = (2.473469 * (ln(X) + 4.447916011)) + (-17.31087 * (sqrt(X) - 0.108180083))  ln(HR) = (2.980902 * (ln(X) + 3.508897203)) + (-7.998696 * (sqrt(X) - 0.1730026094))  ln(HR) = (0.1526095 * (1/sqrt(X) - 5.780259637)) + (0.4977897 * (ln(X) + 3.508897203))  ln(HR) = (-8664.848 * (X^2^ - 0.0001109298)) + (-3526.299 * ((X^2^ * ln(X)) + 0.0005050975))  ln(HR) = (-78.76577 * (X - 0.0105323225 )) + (-50.92199 * ((X*ln(X)) + 0.0479568918 )) | FIB-4 / 100  APRI / 10  (AST/ALT) / 100 |
| Diabetics  FIB-4 Cirrhosis  Death  APRI Cirrhosis  Death  AST/ALT Cirrhosis  Death | ln(HR) = (59.71454 * (sqrt(X) - 0.1190695095)) + (-86.96619 * (X - 0.0141775481))  ln(HR) = (0.0053446 * ((1/X) - 70.53405801)) + (-.2805157 * (1/sqrt(X) - 8.398455692))  ln(HR) = (2.47445 * (ln(X) + 3.359358204)) + (-7.407513 * (X - 0.034757559))  ln(HR) = (-7.65e-06 * ((1/X^2^) - 827.754331)) + (0.0066514 * ((1/X) - 28.77072003))  ln(HR) = (14.90623 * (X - 0.0950818696)) + (-28.40039 * (X^3^ - 0.0008595935))  ln(HR) = (4.037292 * (X - 0.0950818696)) + (-4.97383 * ((X*ln(X)) + 0.223729253)) | FIB-4 / 100  APRI / 10  (AST/ALT) / 10 |
| Obese subjects  FIB-4 Cirrhosis  Death  APRI Cirrhosis  Death  AST/ALT Cirrhosis  Death | ln(HR) = (1.371489 * (sqrt(X) - 0.1065096183)) + (-19.03397 * ((sqrt(X)*ln(X)) + 0.4770608375))  ln(HR) = (2.145916 * (ln(X) + 4.479039971)) + (-13.25871 * (sqrt(X) - 0.1065096183))  ln(HR) = (3.410527 * (ln(X) + 3.450691063)) + (-10.36099 * (sqrt(X) - 0.178111498))  ln(HR) = (0.6875455 * (ln(X) + 3.450691063)) + (.0987988 * (ln(X)^2 - 11.90726881))  ln(HR) = (15.71294 * (X - 0.0920807653)) + (-19.98895 * (X^3^ - 0.0007807406))  ln(HR) = (3.728236 * (X - 0.0920807653)) + (-6.959057 * ((X*ln(X)) + 0.2196208391)) | FIB-4 / 100  APRI / 10  (AST/ALT) / 10 |
| Hazardous alcohol use  FIB-4 Cirrhosis  Death  APRI Cirrhosis  Death  AST/ALT Cirrhosis  Death | ln(HR) = (3.345437 * (sqrt(X) - 0.1182016923)) + (-13.78239 * ((sqrt(X)*ln(X)) + 0.5048070067))  ln(HR) = (-9.367378 * (sqrt(X) - 0.1182016923)) + (-12.91872 * ((sqrt(X)*ln(X)) + 0.5048070067))  ln(HR) = (2.506574 * (ln(X) + 3.094577076)) + (-6.126846 * (sqrt(X) - 0.212824257))  ln(HR) = (0.1591327 * (1/sqrt(X) - 4.698712515)) + (0.4441998 * (ln(X) + 3.094577076))  ln(HR) = (249.1692 * (X - 0.0104756905)) + (-2366.264 * (X^2^ - 0.0001097401))  ln(HR) = (-118.8272 * (X- 0.0104756905)) + (-64.76302 * ((X*ln(X)) + 0.0477555084)) | FIB-4 / 100  APRI / 10  (AST/ALT) / 100 |

Only the four main models are shown here, not those for combinations of risk factors. NB: sqrt() = square root, ln() = natural logarithm

**Table S9: Net benefit and diagnostic results for all three markers at several thresholds, for predicting cirrhosis/HCC within 10 years of first exposure.**

| **Marker/ threshold** | **Marker**  **value*** | **True**  **positive**  **rate** | **False**  **positive**  **rate** | **True**  **negative**  **rate** | **False**  **negative**  **rate** | **Sens** | **Spec** | **Positive**  **Likelihood**  **Ratio** | **Net benefit** | | **Advantage of marker compared to treat-all** | |
| --- | --- | --- | --- | --- | --- | --- | --- | --- | --- | --- | --- | --- |
|  |  |  |  |  |  |  |  |  | Marker | Treat-all | Overall net benefit^6^ | Referrals avoided^7^ |
| FIB-4  Pt>=0.008^1^  Pt>=0.01  Pt>=0.02  Pt>=0.03  Pt>=0.047^2^  Pt>=0.10  Pt>=0.20 | 1.30  1.49  2.13  2.60  3.25  4.68  6.74 | 0.0233  0.0288  0.0587  0.0912  0.1425  0.2409  0.3114 | 0.9767  0.9712  0.9413  0.9088  0.8575  0.7591  0.6886 | 0.9964  0.9960  0.9949  0.9942  0.9934  0.9924  0.9916 | 0.0036  0.0040  0.0051  0.0058  0.0066  0.0076  0.0084 | 0.74  0.67  0.51  0.43  0.35  0.23  0.13 | 0.70  0.78  0.92  0.96  0.98  0.99  1.00 | 2.50  3.08  6.39  10.19  16.79  32.26  46.54 | 0.0047  0.0042  0.0034  0.0029  0.0024  0.0014  0.0006 | 0.0018  -0.0005  -0.0108  -0.0212  -0.0398  -0.1006  -0.2382 | 0.0029  0.0048  0.0141  0.0241  0.0422  0.1020  0.2387 | 37.9  47.2  69.1  77.9  84.8  91.8  95.5 |
| APRI  Pt>=0.008^1^  Pt>=0.01  Pt>=0.02  Pt>=0.03  Pt>=0.10  Pt>=0.16^3^  Pt>=0.20 | 0.29  0.33  0.45  0.54  1.07  1.50  1.80 | 0.0301  0.0398  0.0785  0.1068  0.2013  0.2266  0.2264 | 0.9699  0.9602  0.9215  0.8932  0.7987  0.7734  0.7736 | 0.9970  0.9966  0.9959  0.9954  0.9935  0.9927  0.9922 | 0.0030  0.0034  0.0041  0.0046  0.0065  0.0073  0.0078 | 0.78  0.73  0.66  0.61  0.40  0.29  0.23 | 0.74  0.82  0.92  0.94  0.98  0.99  0.99 | 3.03  3.97  7.75  10.71  23.57  28.28  28.80 | 0.0059  0.0057  0.0054  0.0050  0.0024  0.0010  0.0003 | 0.0018  -0.0005  -0.0108  -0.0212  -0.1006  -0.1808  -0.2382 | 0.0041  0.0063  0.0162  0.0262  0.1030  0.1818  0.2385 | 53.1  62.1  79.3  84.6  92.7  94.7  95.4 |
| AST/ALT  Pt>=0.007^4^  Pt>=0.008^1^  Pt>=0.01  Pt>=0.02  Pt>=0.023^5^  Pt>=0.03  Pt>=0.10  Pt>=0.20^8^ | 1.00  1.05  1.28  1.87  2.00  2.21  3.43  5.00 | 0.0121  0.0129  0.0166  0.0396  0.0438  0.0633  0.1258  0.1064 | 0.9879  0.9871  0.9834  0.9604  0.9562  0.9367  0.8742  0.8936 | 0.9933  0.9933  0.9930  0.9921  0.9919  0.9917  0.9909  0.9906 | 0.0067  0.0067  0.0070  0.0079  0.0081  0.0083  0.0091  0.0094 | 0.65  0.60  0.45  0.20  0.17  0.14  0.04  0.01 | 0.50  0.56  0.75  0.95  0.96  0.98  0.99  1.00 | 1.29  1.38  1.77  4.31  4.80  7.07  15.06  12.47 | 0.0024  0.0023  0.0017  0.0010  0.0008  0.0007  0.0001  -0.0001 | 0.0022  0.0018  -0.0005  -0.0108  -0.0143  -0.0212  -0.1006  -0.2182 | 0.0003  0.0005  0.0022  0.0117  0.0151  0.0219  0.1007  0.2181 | <10  <10  22.0  57.4  62.9  70.7  90.6  94.9 |

NB: True and false positives and negatives are shown as proportions rather than actual numbers.

NB: Sens = sensitivity, Spec = specificity, Positive likelihood ratio = (sensitivity/(1-specificity))

NB: Results take account of the competing risk of death.

^1^ A threshold probability of 0.0077 corresponds with the cut-point of 1.30 on the FIB-4 for ruling out advanced fibrosis, according to EASL.

^2^ A threshold probability of 0.047 corresponds with the commonly utilised cut-point of 3.25 on the FIB-4.

^3^ A threshold probability of 0.1611 corresponds to the commonly utilised cut-point of 1.50 on the APRI.

^4^ A threshold probability of 0.0073 corresponds to the commonly utilised cut-point of 1.0 on the AST/ALT.

^5^ A threshold probability of 0.0234 corresponds to the commonly utilised cut-point of 2.0 on the AST/ALT.

^6^ Overall net benefit compared to treat-all strategy (Overall net benefit = net benefit of marker – net benefit of treat all strategy).

^7^ No. of unnecessary cirrhosis referrals avoided per 100 patients =(net benefit of marker – net benefit of treat all)/(Pt/(1-Pt))*100.

^8^ For the AST/ALT, at high risk levels there are very few cases. The actual risk level utilised here (and the highest risk according to the marker) was ~19%.

**Table S10: Possible case-finding strategies for a 1% risk of cirrhosis/HCC**

| **Population**  **(N screened)** | **Marker** | **Marker score** | **No. referrals** | **Cases**  **detected**  **(%)*** | **Cases missed** | **Total cases** | **Unnecessary referrals** |
| --- | --- | --- | --- | --- | --- | --- | --- |
| Main cohort  (N=100,000) |  |  |  |  |  |  |  |
|  | APRI | 0.33 | 19,020 | 760 (80.0%) | 190 | 950 | 18,270 |
|  | FIB-4 | 1.49 | 22,260 | 640 (67.4%) | 310 | 950 | 21,620 |
|  | AST/ALT | 1.28 | 25,380 | 420 (44.2%) | 530 | 950 | 24,960 |
| Obese patients  (N=24,000) |  |  |  |  |  |  |  |
|  | APRI | 0.32 | 6,220 | 250 (89.3%) | 30 | 280 | 5,980 |
|  | FIB-4 | 1.31 | 6,490 | 220 (78.6%) | 60 | 280 | 6,280 |
|  | AST/ALT | 0.95 | 9,410 | 150 (53.6%) | 130 | 280 | 9,260 |
| Hazardous alcohol use  (N=14,000) |  |  |  |  |  |  |  |
|  | APRI | 0.22 | 8,730 | 420 (93.3%) | 30 | 450 | 8,310 |
|  | FIB-4 | 0.84 | 9,060 | 410 (91.1%) | 40 | 450 | 8,640 |
|  | AST/ALT | 0.62 | 11,830 | 420 (93.3%) | 30 | 450 | 11,410 |
| Diabetic patients  (N=8,800) |  |  |  |  |  |  |  |
|  | APRI | 0.25 | 3,960 | 170 (94.4%) | 10 | 180 | 3,790 |
|  | FIB-4 | 1.16 | 4,470 | 160 (88.9%) | 20 | 180 | 4,310 |
|  | AST/ALT | 0.46 | 8,300 | 170 (94.4%) | 10 | 180 | 8,130 |
| Obese with diabetes  (N=4,200) |  |  |  |  |  |  |  |
|  | APRI | 0.25 | 2,170 | 110 (100%) | 0** | 110 | 2,060 |
|  | FIB-4 | 1.09 | 2,180 | 100 (90.9%) | 10 | 110 | 2,080 |
|  | AST/ALT | 0.54 | 3,610 | 100 (90.9%) | 10 | 110 | 3,510 |
| Obese and hazardous alcohol use  (N=3,900) |  |  |  |  |  |  |  |
|  | APRI | 0.26 | 2,240 | 110 (100%) | 0** | 110 | 2,130 |
|  | FIB-4 | 1.01 | 2,020 | 100 (90.9%) | 10 | 110 | 1,920 |
|  | AST/ALT | 0.65 | 2,810 | 100 (90.9%) | 10 | 110 | 2,710 |
| Diab. and hazardous alcohol use  (N=1,400) |  |  |  |  |  |  |  |
|  | APRI | 0.21 | 1,010 | 60 (100%) | 0** | 60 | 950 |
|  | FIB-4 | 0.99 | 960 | 60 (100%) | 0** | 60 | 900 |
|  | AST/ALT | 0.60 | 1,180 | 60 (100%) | 0** | 60 | 1,120 |
| All three risk factors  (N=700) |  |  |  |  |  |  |  |
|  | APRI | 0.19 | 580 | 40 (100%) | 0** | 40 | 540 |
|  | FIB-4 | 1.02 | 460 | 40 (100%) | 0** | 40 | 420 |
|  | AST/ALT | 0.55 | 610 | 40 (100%) | 0** | 40 | 570 |

* The percentage is out of the total number of cases.

** Due to small numbers these cells have been set to zero in the above table. Note also that we cannot claim our model counts to be accurate down to the single subject, so all counts have been rounded to the nearest 10. As a result, some row totals may be slightly out by 10. This has not affected comparisons between the markers in each risk group, however.

**Table S11: Possible case-finding strategies for a 10% risk of cirrhosis/HCC**

| **Population**  **(N screened)** | **Marker** | **Marker score** | **No. referrals** | **Cases**  **detected**  **(%)*** | **Cases missed** | **Total cases** | **Unnecessary referrals** |
| --- | --- | --- | --- | --- | --- | --- | --- |
| Main cohort  (N=100,000) |  |  |  |  |  |  |  |
|  | APRI | 1.07 | 2,110 | 420 (44.2%) | 530 | 950 | 1,680 |
|  | FIB-4 | 4.68 | 910 | 220 (23.2%) | 730 | 950 | 690 |
|  | AST/ALT | 3.43 | 290 | 40 (4.2%) | 910 | 950 | 250 |
| Obese patients  (N=24,000) |  |  |  |  |  |  |  |
|  | APRI | 0.93 | 700 | 140 (50.0%) | 140 | 280 | 560 |
|  | FIB-4 | 3.50 | 370 | 90 (32.1%) | 190 | 280 | 290 |
|  | AST/ALT | 2.97 | 70 | 10 (3.6%) | 270 | 280 | 60 |
| Hazardous alcohol use  (N=14,000) |  |  |  |  |  |  |  |
|  | APRI | 0.79 | 1,200 | 310 (68.9%) | 140 | 450 | 880 |
|  | FIB-4 | 2.88 | 870 | 240 (53.3%) | 210 | 450 | 630 |
|  | AST/ALT | 1.94 | 590 | 130 (28.9%) | 320 | 450 | 460 |
| Diabetic patients  (N=8,800) |  |  |  |  |  |  |  |
|  | APRI | 0.74 | 550 | 110 (60.3%) | 70 | 180 | 440 |
|  | FIB-4 | 3.37 | 300 | 60 (33.5%) | 120 | 180 | 240 |
|  | AST/ALT | 2.99 | 30 | 0** (0%) | 180 | 180 | 30 |
| Obese with diabetes  (N=4,200) |  |  |  |  |  |  |  |
|  | APRI | 0.69 | 380 | 80 (72.7%) | 30 | 110 | 300 |
|  | FIB-4 | 2.71 | 240 | 50 (45.5%) | 60 | 110 | 190 |
|  | AST/ALT | 2.45 | 20 | 0** (0%) | 110 | 110 | 20 |
| Obese and hazardous alcohol use  (N=3,900) |  |  |  |  |  |  |  |
|  | APRI | 0.79 | 330 | 80 (72.7%) | 30 | 110 | 250 |
|  | FIB-4 | 2.52 | 280 | 70 (63.6%) | 40 | 110 | 210 |
|  | AST/ALT | 1.61 | 150 | 30 (27.3%) | 80 | 110 | 120 |
| Diab. and hazardous alcohol use  (N=1,400) |  |  |  |  |  |  |  |
|  | APRI | 0.62 | 230 | 50 (83.3%) | 10 | 60 | 170 |
|  | FIB-4 | 2.40 | 180 | 40 (66.7%) | 20 | 60 | 150 |
|  | AST/ALT | 1.44 | 120 | 20 (33.3%) | 40 | 60 | 110 |
| All three risk factors  (N=700) |  |  |  |  |  |  |  |
|  | APRI | 0.55 | 170 | 40 (100%) | 0** | 40 | 130 |
|  | FIB-4 | 1.92 | 160 | 30 (75.0%) | 10 | 40 | 130 |
|  | AST/ALT | 1.16 | 100 | 20 (50.0%) | 20 | 40 | 80 |

* The percentage is out of the total number of cases.

** Due to small numbers these cells have been set to zero in the above table. Note also that we cannot claim our model counts to be accurate down to the single subject, so all counts have been rounded to the nearest 10. As a result, some row totals may be slightly out by 10. This has not affected comparisons between the markers in each risk group, however.

**Table S12: Calculation of size of each pseudo-population**

| **Population** | **N** | **Numbers shown per 2 sig. figs.** | **Table 5 numbers shown per** |
| --- | --- | --- | --- |
| Main cohort | 203,005 | (203,005/203,005) = 1.0 | 1 * 100,000 = 100,000 |
| Obese (high BMI) | 47,818 | (47818/203005) = 0.2356 =~0.24 | 0.24 * 100,000 = 24,000 |
| Diabetes | 17,784 | (17784/203005) = 0.0876 =~0.088 | 0.088 * 100,000 = 8,800 |
| Hazardous alcohol | 29,139 | (29139/203005) = 0.1435 =~0.14 | 0.14 * 100,000 = 14,000 |
| Alcohol and obese | 7,919 | (7919/203005) = 0.039 =~0.039 | 0.039 * 100,000 = 3,900 |
| Diabetes and obese | 8,454 | (8454/203005) = 0.0416 =~0.042 | 0.042 * 100,000 = 4,200 |
| Diabetes and alcohol | 2,872 | (2872/203005) = 0.0141 =~ 0.014 | 0.014 * 100,000 = 1,400 |
| All 3 risk factors | 1,429 | (1429/203005) = 0.0070 =~ 0.007 | 0.007 * 100,000 = 700 |

NB: Population sizes have been chosen to 2 significant figures each time.

**Table S13: Possible case-finding strategies for a 3% risk of cirrhosis/HCC, cause-specific analysis (does not take account of competing risk of death)**

| Population  (N screened) | Marker | Marker score | No. referrals | Cases  detected  (%)* | Cases missed | Total cases | Unnecessary referrals |
| --- | --- | --- | --- | --- | --- | --- | --- |
| Main cohort  (N=100,000) |  |  |  |  |  |  |  |
|  | APRI | 0.51 | 6,950 | 790 (75.2%) | 260 | 1,050 | 6,160 |
|  | FIB-4 | 2.25 | 7,120 | 650 (61.9%) | 400 | 1,050 | 6,470 |
|  | AST/ALT | 2.02 | 2,970 | 200 (19.0%) | 850 | 1,050 | 2,770 |
| Obese patients  (N=24,000) |  |  |  |  |  |  |  |
|  | APRI | 0.48 | 2,520 | 250 (83.3%) | 50 | 300 | 2,280 |
|  | FIB-4 | 1.91 | 2,380 | 200 (66.7%) | 100 | 300 | 2,190 |
|  | AST/ALT | 1.65 | 970 | 60 (20.0%) | 240 | 300 | 910 |
| Hazardous alcohol use  (N=14,000) |  |  |  |  |  |  |  |
|  | APRI | 0.38 | 3,650 | 430 (89.6%) | 50 | 480 | 3,220 |
|  | FIB-4 | 1.43 | 4,050 | 410 (85.4%) | 80 | 480 | 3,650 |
|  | AST/ALT | 1.13 | 4,560 | 320 (66.7%) | 160 | 480 | 4,240 |
| Diabetic patients  (N=8,800) |  |  |  |  |  |  |  |
|  | APRI | 0.37 | 1,930 | 200 (90.9%) | 20 | 220 | 1,740 |
|  | FIB-4 | 1.71 | 2,060 | 170 (77.3%) | 40 | 220 | 1,880 |
|  | AST/ALT | 1.15 | 2,080 | 80 (36.4%) | 140 | 220 | 2,000 |
| Obese with diabetes  (N=4,200) |  |  |  |  |  |  |  |
|  | APRI | 0.37 | 1,180 | 120 (100%) | 0** | 120 | 1,060 |
|  | FIB-4 | 1.54 | 1,120 | 110 (91.7%) | 10 | 120 | 1,010 |
|  | AST/ALT | 0.94 | 1,460 | 70 (58.3%) | 50 | 120 | 1,390 |
| Obese and hazardous alcohol use  (N=3,900) |  |  |  |  |  |  |  |
|  | APRI | 0.42 | 1,020 | 110 (91.7%) | 10 | 120 | 910 |
|  | FIB-4 | 1.47 | 1,030 | 100 (83.3%) | 20 | 120 | 930 |
|  | AST/ALT | 1.01 | 990 | 70 (58.3%) | 50 | 120 | 920 |
| Diab. and hazardous alcohol use  (N=1,400) |  |  |  |  |  |  |  |
|  | APRI | 0.32 | 600 | 70 (100%) | 0** | 70 | 530 |
|  | FIB-4 | 1.39 | 600 | 70 (100%) | 0** | 70 | 530 |
|  | AST/ALT | 0.82 | 750 | 60 (85.7%) | 10 | 70 | 690 |
| All three risk factors  (N=700) |  |  |  |  |  |  |  |
|  | APRI | 0.29 | 400 | 40 (100%) | 0** | 40 | 360 |
|  | FIB-4 | 1.27 | 340 | 40 (100%) | 0** | 40 | 300 |
|  | AST/ALT | 0.73 | 410 | 40 (100%) | 0** | 40 | 370 |

* The percentage is out of the total number of cases.

** Due to small numbers these cells have been set to zero in the above table. Note also that we cannot claim our model counts to be accurate down to the single subject, so all counts have been rounded to the nearest 10. As a result, some row totals may be slightly out by 10. This has not affected comparisons between the markers in each risk group, however.

**Table S14: Equivalent marker values associated with each threshold, by age group**

| **Commonly applied marker cut-point** | **Age group** | **Risk threshold** | **FIB-4 score** | **APRI score** | **AST/ALT score** |
| --- | --- | --- | --- | --- | --- |
| AST/ALT>=1.00  Cirrhosis | <65  >=65 | 0.008  0.010 | 1.05  1.78 | 0.30  0.29 | 1.00 (Cirrhosis)  1.00 (Cirrhosis) |
| FIB-4 >=1.30  EASL | <65  >=65 | 0.014  0.006 | 1.30 (EASL)  1.30 (EASL) | 0.38  0.21 | 1.42  0.03 |
| AST/ALT>=2.00  ALD | <65  >=65 | 0.036  0.019 | 1.88  2.38 | 0.58  0.40 | 2.00 (ALD)  2.00 (ALD) |
| FIB-4 >=2.67  NAFLD | <65  >=65 | 0.088  0.025 | 2.67 (NAFLD)  2.67 (NAFLD) | 0.92  0.46 | 2.58  2.31 |
| FIB-4>=3.25  Cirrhosis | <65  >=65 | 0.142  0.041 | 3.25 (Cirrhosis)  3.25 (Cirrhosis) | 1.23  0.59 | 2.93  2.89 |
| APRI >=1.50  Cirrhosis | <65  >=65 | 0.190  0.196 | 3.70  6.05 | 1.50 (Cirrhosis)  1.50 (Cirrhosis) | 3.15  3.86 |

Note that these marker cut-points have been identified in different populations and risk groups and are included here merely to highlight the difference in risk threshold that exists between the two age groups, if a specific marker score were to be implemented.

**Table S15: Harrell’s concordance C-statistic for each fibrosis marker, for different periods of follow-up time**

|  | **Period of follow-up time (no. of subjects)** | | | | |
| --- | --- | --- | --- | --- | --- |
| **Marker** | All time  (10 years total)  (N=203,005) | 1 year  onwards  (9 years total)  (N=183,479) | 3 years  onwards  (7 years total)  (N=161,162) | 5 years  onwards  (5 years total)  (N=146,449) | 8 years  onwards  (2 years total)  (N=114,131) |
| FIB-4  Harrell’s C-index | 0.83 | 0.81 | 0.79 | 0.77 | 0.75 |
| APRI  Harrell’s C-index | 0.84 | 0.82 | 0.80 | 0.79 | 0.81 |
| AST/ALT  Harrell’s C-index | 0.67 | 0.65 | 0.62 | 0.59 | 0.55 |

Note: to achieve these results the C-statistic has first been calculated for the main model including all follow-up time. For the other time periods, the entry time to the study has been moved forwards by 1, 3, 5, and 8 years, and the statistic recalculated. This has not required the Cox model to be reran, so the results are with respect to the same prognostic model, using the same fractional polynomial terms. NB: Numbers do not take account of competing risk of death.

**Table S16: Comparing number of referrals at the 3% risk threshold for the main analysis with symptomatic codes only***

| **Outcome codes included** | **Marker** | **Marker score** | **No. referrals** | **Cases**  **detected**  **(%)**** | **Cases missed** | **Total cases** | **Unnecessary referrals** |
| --- | --- | --- | --- | --- | --- | --- | --- |
| All codes |  |  |  |  |  |  |  |
|  | APRI | 0.51 | 6,950 | 790 (75.2%) | 260 | 1,050 | 6,160 |
|  | FIB-4 | 2.25 | 7,120 | 650 (61.9%) | 400 | 1,050 | 6,470 |
|  | AST/ALT | 2.02 | 2,970 | 200 (19.0%) | 850 | 1,050 | 2,770 |
| Symptomatic codes only |  |  |  |  |  |  |  |
|  | APRI | 0.84 | 2,908 | 251 (51.3%) | 238 | 489 | 2,657 |
|  | FIB-4 | 3.14 | 2,608 | 211 (43.1%) | 278 | 489 | 2,397 |
|  | AST/ALT | 2.68 | 795 | 43 (8.8%) | 446 | 489 | 752 |

* Symptomatic codes include liver cell carcinoma, oesophageal varices with bleeding, portal hypertension, chronic hepatic failure, hepatorenal syndrome and trans-jugular intrahepatic insertion of stent/graft. ** The percentage is out of the total number of cases.

NB: Numbers do not take account of competing risk of death.

**Table S17: Numbers of cirrhosis/HCC cases remaining for analysis after applying different exclusion periods**

| **Population** | **Total N included in analysis** | **Number of cirrhosis/HCC cases within 10 years** | **Total number of subjects dropped** |
| --- | --- | --- | --- |
| Starting sample | 204,871 | 1,693 | - |
| 30-day exclusion | 203,005 | 1,583  (n=110 cases dropped) | 1,866 |
| 60-day exclusion | 200,881 | 1,529  (n=164 cases dropped) | 3,990 |
| 90-day exclusion | 198,808 | 1,487  (n=206 cases dropped) | 6,063 |
| 120-day exclusion | 196,887 | 1,459  (n=234 cases dropped) | 7,984 |
| 150-day exclusion | 195,180 | 1,436  (n=257 cases dropped) | 9,691 |
| 180-day exclusion | 193,429 | 1,413  (n=280 cases dropped) | 11,442 |

Note that for the original analysis the at-risk period starts 30 days after the exposure date.

The same approach has been taken with the other examples.

**Table S18: Comparison of numbers of referrals per 100,000 for 30-day and 180-day exclusion periods**

| **Exclusion period** | **Marker** | **Marker score** | **No. referrals*** | **Cases**  **detected**  **(%)**** | **Cases missed** | **Total cases** | **Unnecessary referrals** |
| --- | --- | --- | --- | --- | --- | --- | --- |
| 30-days |  |  |  |  |  |  |  |
|  | APRI | 0.51 | 6,950 | 790 (75.2%) | 260 | 1,050 | 6,160 |
|  | FIB-4 | 2.25 | 7,120 | 650 (61.9%) | 400 | 1,050 | 6,470 |
|  | AST/ALT | 2.02 | 2,970 | 200 (19.0%) | 850 | 1,050 | 2,770 |
| 180-days |  |  |  |  |  |  |  |
|  | APRI | 0.52 | 6,185 | 683 (69.1%) | 306 | 989 | 5,502 |
|  | FIB-4 | 2.27 | 6,519 | 576 (58.2%) | 413 | 989 | 5,943 |
|  | AST/ALT | 2.10 | 2,502 | 149 (15.1%) | 840 | 989 | 2,353 |

* For a 3% risk of cirrhosis/HCC

** The percentage is out of the total number of cases.

NB: Numbers do not take account of competing risk of death.

**Table S19: Demographic characteristics of analysis cohort compared with excluded subjects**

| **Characteristic** | **All patients** | **Excluded from analysis** | **Included in analysis*** |
| --- | --- | --- | --- |
| Total N | 2,145,178 (100%) | 1,942,173 (90.54%) | 203,005 (9.46%) |
| Sex  Male  Female | 951,926 (44.38%)  1,193,252 (55.62%) | 863,657 (44.47%)  1,078,516 (55.53%) | 88,269 (43.48%)  114,736 (56.52%) |
| Diagnosed with cirrhosis/ HCC  Yes  No | 20,734 (0.97%)  2,124,444 (99.03%) | 18,542 (0.95%)  1,923,631 (99.05%) | 2,192 (1.08%)**  200,813 (98.92%) |
| Diagnosed with HCC  Yes  No | 1,890 (0.09%)  2,143,288 (99.91%) | 1,674 (0.09%)  1,940,499 (99.91%) | 216 (0.11%)  202,789 (99.89%) |
| Deprivation quintile***  Most deprived  Next most deprived  Median deprivation  Next least deprived  Least deprived  Missing | 434,935 (20.28%)  427,353 (19.92%)  442,177 (20.61%)  391,133 (18.23%)  421,157 (19.63%)  28,423 (1.32%) | 400,337 (20.61%)  381,859 (19.66%)  395,368 (20.36%)  342,669 (17.64%)  396,467 (20.41%)  25,473 (1.31%) | 34,598 (17.04%)  45,494 (22.41%)  46,809 (23.06%)  48,464 (23.87%)  24,690 (12.16%)  2,950 (1.45%) |
| Age when cirrhosis first diagnosed (years)  No cirrhosis diagnosis  <18  18-39  40-59  60-79  80+ | 2,124,444 (99.03%)  81 (0%)  1,696 (0.08%)  7,861 (0.37%)  8,870 (0.41%)  2,226 (0.10%)**** | 1,923,631 (99.05%)  81 (0%)  1,568 (0.08%)  7,097 (0.37%)  7,831 (0.40%)  1,965 (0.10%) | 200,813 (98.92%)  -  128 (0.06%)  764 (0.38%)  1,039 (0.51%)  261 (0.13%) |

* Patients with all three fibrosis markers who are included in the main analysis.

** Includes all cirrhosis cases, whether or not they made it into the final analysis.

*** For deprivation we chose the level closest to the study start date (1/1/2000) rather than the exposure date as for the main analysis, as most patients do not have an exposure date.

**** Includes a small number of cirrhosis cases (n<5) with missing diagnosis dates.

**Table S20: Incidence rates by demographic characteristic, for patients without a FIB-4 omitted from the main analysis**

|  | **N patients** | **Person years** | **Failures**  **(cirrhosis/HCC)** | **Incidence rate of cirrhosis/HCC (per 1000 person-years)** | **Cumulative 10-year risk**  **/ incidence proportion (%)*** |
| --- | --- | --- | --- | --- | --- |
| All patients  Male  Female | 1,858,852 (100.0%)  823,678 (44.3%)  1,035,174 (55.7%) | 12,366,607.94  5,367,954.84  6,998,653.10 | 9,049  5,689  3,360 | 0.73 (0.72 – 0.75)  1.06 (1.03 – 1.09)  0.48 (0.46 – 0.50) | 0.68 (0.67 – 0.70)  0.98 (0.96 – 1.01)  0.45 (0.44 – 0.47) |
| Age when tested  18 – 39  40 – 59  60 – 79  80+ | 692,825 (37.3%)  612,076 (32.9%)  441,231 (23.7%)  112,720 (6.1%) | 4,186,655.00  4,546,659.09  3,128,652.46  504,641.38 | 1,079  4,104  3,388  478 | 0.26 (0.24 – 0.27)  0.90 (0.88 – 0.93)  1.08 (1.05 – 1.12)  0.95 (0.87 – 1.04) | 0.27 (0.25 – 0.29)  0.88 (0.86 – 0.91)  0.94 (0.91 – 0.97)  0.49 (0.45 – 0.54) |
| Deprivation quintile  Most deprived  Next most deprived  Median deprivation  Next least deprived  Least deprived  Missing | 370,119 (19.9%)  365,861 (19.7%)  375,613 (20.2%)  332,989 (17.9%)  396,983 (21.4%)  17,287 (0.9%) | 2,462,747.46  2,438,567.45  2,477,742.21  2,177,630.17  2,730,902.99  79,017.65 | 2,433  2,042  1,728  1,292  1,496  58 | 0.99 (0.95 – 1.03)  0.84 (0.80 – 0.87)  0.70 (0.67 – 0.73)  0.59 (0.56 – 0.63)  0.55 (0.52 – 0.58)  0.73 (0.57 – 0.95) | 0.91 (0.87 – 0.95)  0.78 (0.75 – 0.82)  0.64 (0.61 – 0.68)  0.56 (0.53 – 0.59)  0.52 (0.50 – 0.55)  0.63 (0.48 – 0.83) |

NB: Incidence rate per 1000 person years is calculated as follows: [(number of failures) / (person years)] * 1000

* Calculated using the stcompet function in Stata, which provides a non-parametric estimate of the cumulative incidence by strata and does not utilise the fractional polynomial method.

Number of patients included = 1,942,173 total excluded (see Table S12), - 1,659 diagnosed before age 18 or before 1/1/2000, -1,866 with a FIB-4 but dropped due to survival time within first 30 days of main analysis, -64,933 patients without GP registration data falling within the study period, -2,218 diagnosed with cirrhosis before blood test date, -166 study exit and entry date are the same, -7 patients who die before date of blood test, -12,472 subjects with a survival time within the first 30 days. Final total patients included in this analysis = 1,858,852. The deprivation quintile assigned to each subject is the one closest to the blood test (entry) date.

30-day exclusion period

Outcome/censoring occurs within 30 days of exposure.

Patients followed-up until cirrhosis/HCC diagnosis, censoring, or the end of the 10-year period.

Assessment of risk factors

Diabetes, alcohol and BMI

Follow-up period of 10 years

(Analysis period)

End of analysis

(10 years after Start date)

Start of analysis

(Start date)

Fibrosis marker

(Exposure date)

N = 203,005

Time

**Figure S1: Study design diagram**


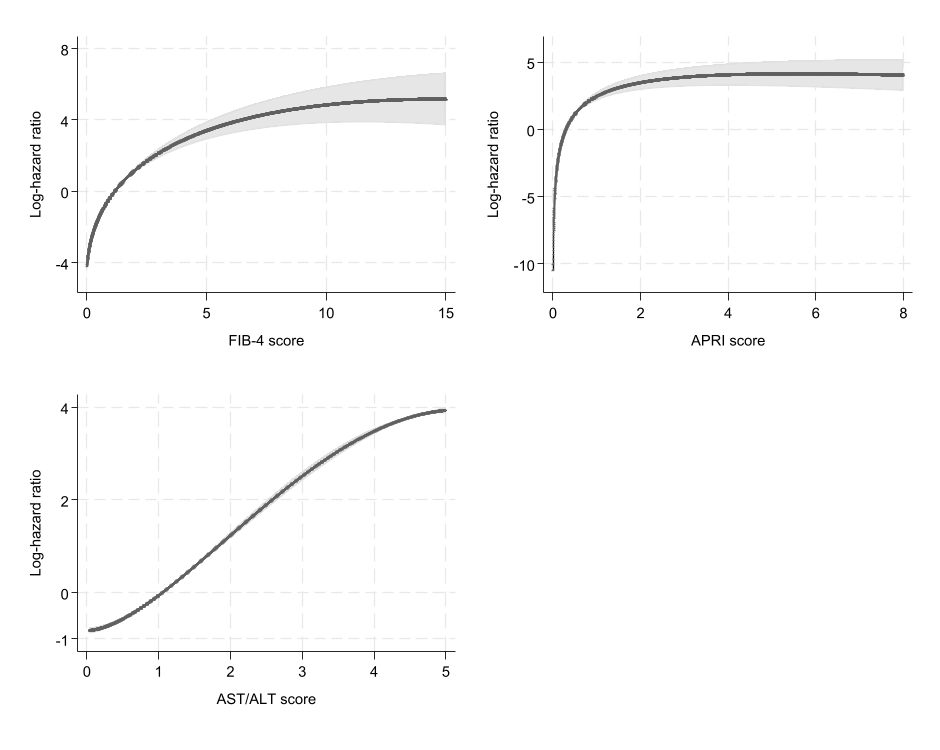


**Figure S2: Log-hazard ratio with 95% confidence region vs. each fibrosis marker score**

NB: The 95% confidence regions are shown by the shaded area around each line.


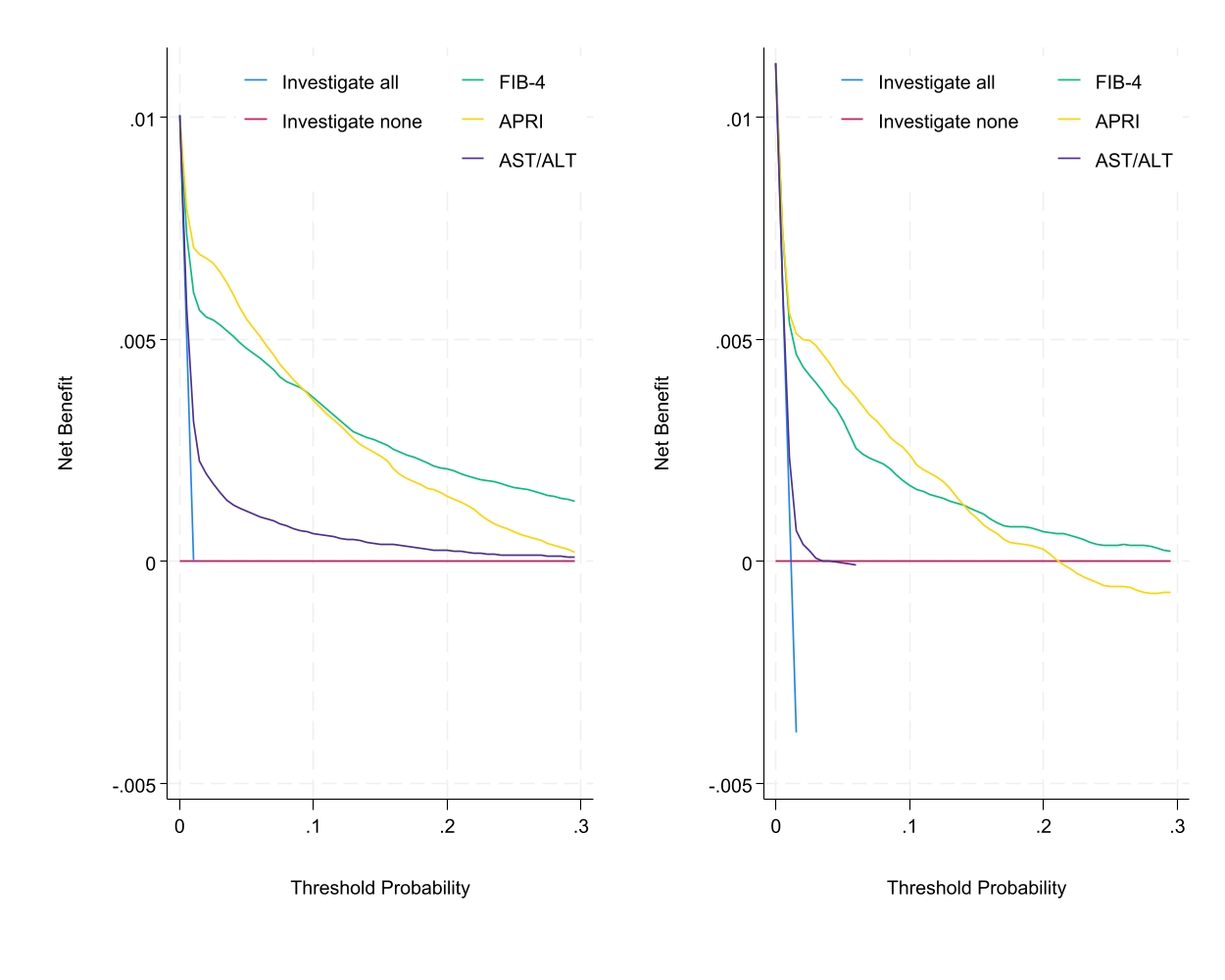


NB: N=134,071 subjects aged <65 and N=68,934 aged >=65, (N=203,005 total)

Note also that this analysis does not take account of competing risk of death.

**Figure S3: DCA plots comparing net benefit in subjects aged <65 years (left) with those aged >=65 years (right)**


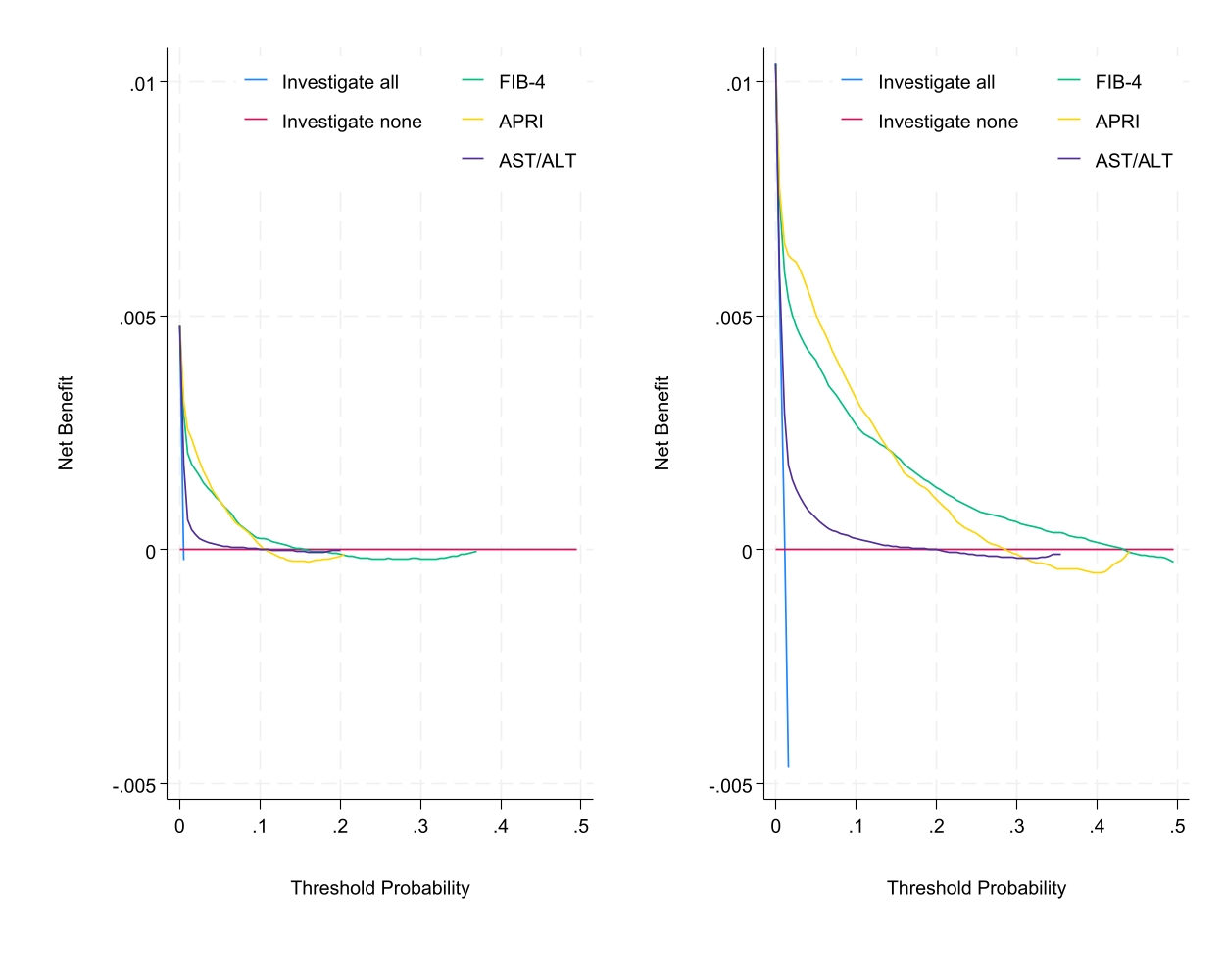


NB: N=203,005 subjects in both analyses but the left-hand plot includes fewer cases; 727 vs 1,583.

Note also that this analysis does not take account of competing risk of death.

**Figure S4: Comparing the DCA curve for the outcome restricted to symptomatic cirrhosis codes (left) with the original analysis including all cirrhosis codes (right).**


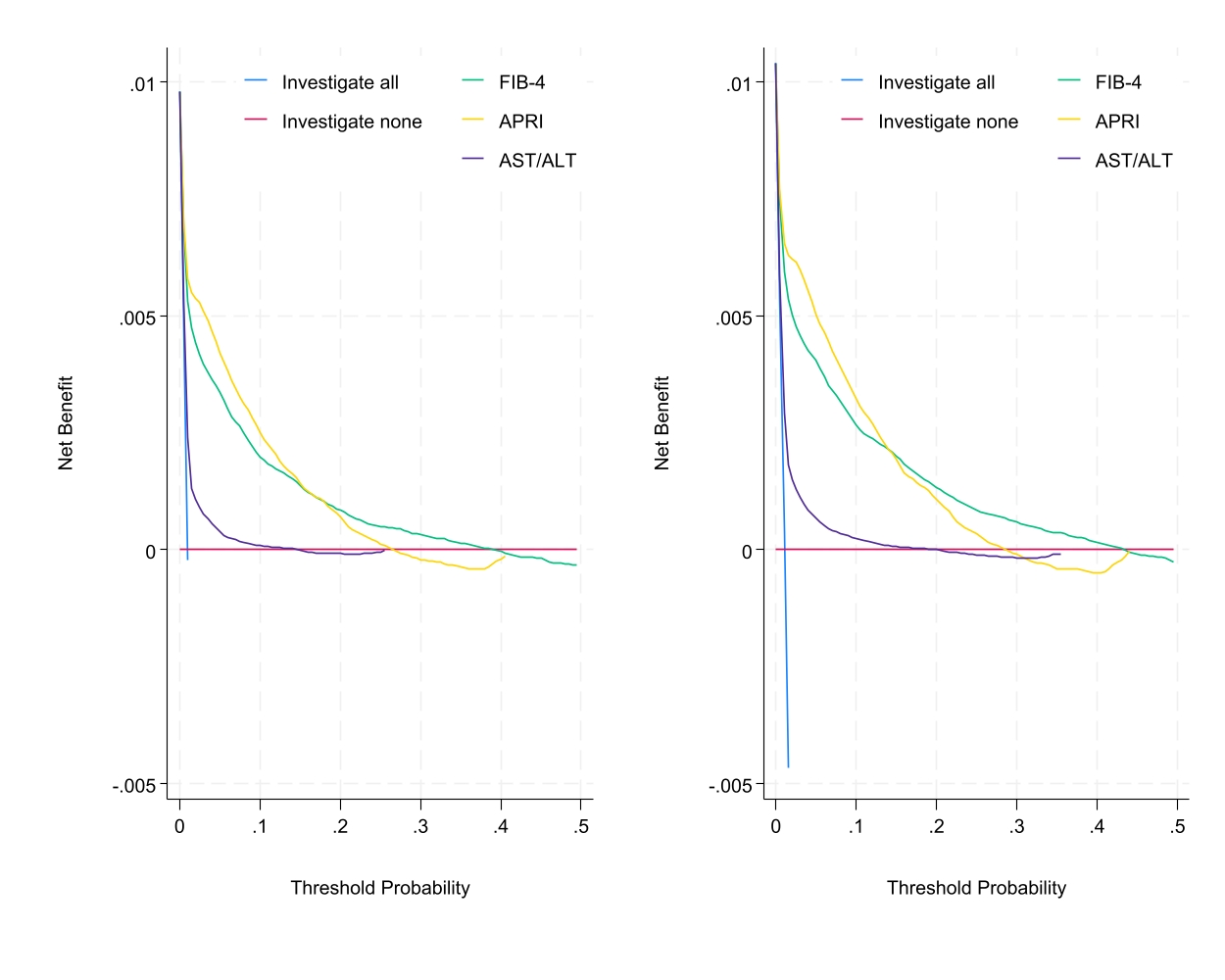


NB: N=193,429 subjects left-hand plot, and N=203,005 subjects right-hand plot.

Note also that this analysis does not take account of competing risk of death.

**Figure S5: DCA curve comparing net benefit for a 180-day exclusion period (left) with the original 30-day exclusion period (right).**
